# A Double-Blinded, Randomized Comparison of Medetomidine-Tiletamine-Zolazepam and Dexmedetomidine-Tiletamine-Zolazepam Anesthesia in Free-Ranging Brown Bears (*Ursus Arctos*)

**DOI:** 10.1371/journal.pone.0170764

**Published:** 2017-01-24

**Authors:** Núria Fandos Esteruelas, Marc Cattet, Andreas Zedrosser, Gordon B. Stenhouse, Susanne Küker, Alina L. Evans, Jon M. Arnemo

**Affiliations:** 1 Department of Forestry and Wildlife Management, Inland Norway University of Applied Sciences, Campus Evenstad, Elverum, Norway; 2 RGL Recovery Wildlife Health & Veterinary Services, Saskatoon, Saskatchewan, Canada; 3 Department of Veterinary Pathology, University of Saskatchewan, Saskatoon, Saskatchewan, Canada; 4 Department of Environmental and Health Studies, University College of Southeast Norway, Porsgrunn, Norway; 5 Department of Integrative Biology and Biodiversity Research, University of Natural Resources and Applied Life Sciences, Vienna, Austria; 6 fRI Research, Hinton, Alberta, Canada; 7 Department of Wildlife, Fish and Environmental Studies, Swedish University of Agricultural Sciences, Umeå, Sweden; University of Bari, ITALY

## Abstract

We compared anesthetic features, blood parameters, and physiological responses to either medetomidine-tiletamine-zolazepam or dexmedetomidine-tiletamine-zolazepam using a double-blinded, randomized experimental design during 40 anesthetic events of free-ranging brown bears (*Ursus arctos*) either captured by helicopter in Sweden or by culvert trap in Canada. Induction was smooth and predictable with both anesthetic protocols. Induction time, the need for supplemental drugs to sustain anesthesia, and capture-related stress were analyzed using generalized linear models, but anesthetic protocol did not differentially affect these variables. Arterial blood gases and acid-base status, and physiological responses were examined using linear mixed models. We documented acidemia (pH of arterial blood < 7.35), hypoxemia (partial pressure of arterial oxygen < 80 mmHg), and hypercapnia (partial pressure of arterial carbon dioxide ≥ 45 mmHg) with both protocols. Arterial pH and oxygen partial pressure were similar between groups with the latter improving markedly after oxygen supplementation (p < 0.001). We documented dose-dependent effects of both anesthetic protocols on induction time and arterial oxygen partial pressure. The partial pressure of arterial carbon dioxide increased as respiratory rate increased with medetomidine-tiletamine-zolazepam, but not with dexmedetomidine-tiletamine-zolazepam, demonstrating a differential drug effect. Differences in heart rate, respiratory rate, and rectal temperature among bears could not be attributed to the anesthetic protocol. Heart rate increased with increasing rectal temperature (p < 0.001) and ordinal day of capture (p = 0.002). Respiratory rate was significantly higher in bears captured by helicopter in Sweden than in bears captured by culvert trap in Canada (p < 0.001). Rectal temperature significantly decreased over time (p ≤ 0.05). Overall, we did not find any benefit of using dexmedetomidine-tiletamine-zolazepam instead of medetomidine-tiletamine-zolazepam in the anesthesia of brown bears. Both drug combinations appeared to be safe and reliable for the anesthesia of free-ranging brown bears captured by helicopter or by culvert trap.

## Introduction

Capture, and anesthesia of wild mammals are required for conservation, research and management purposes [1–3]. The use of anesthetic drugs helps to reduce the stress and pain caused by capture and handling, while providing safety for capture personnel [4]. Brown bears (*Ursus arctos*) have been anesthetized for management and conservation throughout their global range using a variety of anesthetic agents. The most common protocols have combined a dissociative agent with a benzodiazepine or an alpha-2 adrenoceptor agonist [5, 6].

Tiletamine, a dissociative anesthetic, combined in equal parts by weight with zolazepam, a benzodiazepine agonist, has been used for many years in the anesthesia of brown bears, especially in North America [6]. Tiletamine-zolazepam (TZ) produces reliable anesthesia in bears, has a wide safety margin, and causes minimal depression of the cardiovascular and respiratory systems [7, 8]. However, use of TZ requires large drug volumes, provides poor visceral analgesia, and cannot be antagonized [6]. Another concern is extended recovery times, especially when additional (top-up) doses of TZ are administered, exposing anesthetized bears to the risks of inclement weather and predation [9, 10].

Combining TZ with medetomidine (M), an alpha-2 adrenoceptor agonist, counteracts some of the undesired effects of TZ. Medetomidine-tiletamine-zolazepam (MTZ) can be delivered at approximately 25% of the volume of TZ alone [8]. Additionally, M improves analgesia and reduces the effective TZ dose level (mg/kg) required by 75%. The effects of M can be specifically antagonized by atipamezole, an alpha-2 adrenoceptor antagonist [7], making MTZ a “partially reversible” anesthetic protocol.

Medetomidine is a potent, selective, and specific alpha-2 adrenoceptor agonist composed by equal parts of two optical enantiomers, dexmedetomidine and levomedetomidine [11]. The pharmacological effects of M are due almost exclusively to dexmedetomidine [12, 13]. Levomedetomidine is considered an inactive ingredient [12], but may act as a weak partial alpha-2 adrenoceptor agonist or as an inverse alpha-2 adrenoceptor agonist [14], producing opposite sedative and analgesic effects [13, 15].

Dexmedetomidine (D), the dextrorotatory enantiomer, has been used in recent years in the anesthesia of a few wildlife species, including bears [16–20]. Dexmedetomidine combined with TZ (DTZ) has been suggested to cause less respiratory depression than MTZ in bears potentially offering a benefit of using D instead of M [21, 22].

Our study goal was to determine whether DTZ offers any advantage over MTZ in the anesthesia of free-ranging brown bears by comparing induction times, the need for supplemental drugs to sustain anesthesia, stress as quantified by serum cortisol concentrations, arterial blood gases, acid-base status, and physiological responses between anesthetic protocols. To our knowledge, this is the first double-blinded, randomized comparison of the effects of DTZ and MTZ in ursids. We hypothesized that:

1. Induction time—The induction of anesthesia occurs faster with DTZ than with MTZ.
  Quick inductions reduce the potential for physical injury and physiological stress. Shorter induction times have been reported in golden-headed lion tamarins (*Leontopithecus chrysomelas*) anesthetized with D-ketamine compared to M-ketamine [16].
2. Duration of anesthesia—The need for supplemental drugs to sustain anesthesia is lower with DTZ than MTZ.
  Drugs used in wildlife anesthesia should provide enough depth and duration of anesthesia to perform all planned handling procedures without the administration of supplemental (also referred to as top-up) drugs. Further, the supplemental administration of TZ may result in prolonged recoveries [9, 10]. Studies have discovered a longer lasting anesthetic effect of D over M [16].
3. Stress—Stress in response to capture and handling is lower with DTZ than MTZ.
  Blood concentrations of cortisol, and glucose to a lesser extent, are widely-used parameters to assess the stress response to capture and handling in free-ranging wild animals [23, 24]. Medetomidine has been shown to cause greater increases in serum glucose concentration than D [25]. Although the effects of alpha-2 adrenoceptor agonists on cortisol concentrations are controversial [26, 27], we hypothesized that serum concentrations of cortisol, as an indicator of stress, would be less with DTZ.
4. Arterial blood gases and acid-base status—Bears anesthetized with DTZ have higher pH and partial pressure of arterial oxygen (PaO_2_), and lower partial pressure of arterial carbon dioxide (PaCO_2_) than bears anesthetized with MTZ.
  Hypoxemia (PaO_2_ < 80 mmHg) is a common finding in bears anesthetized with MTZ [28, 29]. DTZ, however, was reported to not cause hypoxemia in a study of brown bears [21]. Although pH and blood gases are not routinely recorded in wildlife studies, they provide a valuable physiological assessment of an animal's response to capture and anesthesia.
5. Physiological responses—DTZ produces less cardio-respiratory depression and quicker recovery of normal body temperature than MTZ.
  Ideally, anesthetic drugs should cause minimal depression of the cardiovascular and respiratory systems, and should not suppress the dissipation of excess body heat caused by physical exertion and stress. Several studies have suggested that D has minimal effects on these physiological variables [18, 21].

## Material and Methods

### Scandinavian Brown Bear Research Project (SBBRP)

We captured 31 individual free-ranging brown bears on 34 occasions in Dalarna County, Sweden (61.219756–61.579688 N, 13.019778–15.416586 E) in April-July 2014 and April-May 2015. We applied a randomized, double-blinded design in which 15 individuals were allocated to the MTZ group and 16 to the DTZ group. Three bears were captured twice, once per year, with one bear receiving MTZ followed by DTZ, another receiving DTZ followed by MTZ, and the third receiving DTZ both years. Consequently, the MTZ group comprised 16 anesthetic events and the DTZ group comprised 18 anesthetic events. When two or more bears were together at the time of capture (i.e., family groups), we randomly used one of the study drug combinations for the first bear and alternated the drug for the accompanying bear(s). Captured bears in this study were composed of 16 males and 15 females with 19 bears captured as yearlings, nine bears captured as two year olds, and three captured at both ages. We did not capture larger bears because our dart volumes were limited to ≤3 ml and because access to D in Sweden was limited to a low concentration (0.5 mg/ml) drug solution.

For yearlings, we prepared MTZ by adding 5 mg of M (Domitor^®^ 1 mg/ml per 10 ml per vial, Orion Pharma Animal Health, Turku, Finland) to a vial of TZ (Zoletil^®^ 500 mg/vial, Virbac, Carros, France). We split the solution into six 1.5 ml darts, each dart containing 0.83 mg of M and 83.3 mg of TZ. The remaining 5 mg of M were equally divided and added to each dart (0.83 mg of M per dart). The final solution contained 1.66 mg of M and 83.3 mg of TZ in each dart, with a M:TZ ratio of 1:50. We prepared DTZ in the same way as described above adding 2.5 mg of D (Dexdomitor^®^ 0.5 mg/ml per 10 ml per vial, Orion Pharma Animal Health) to a vial of TZ. We split the solution into six darts, each dart containing 0.415 mg of D and 83.3 mg of TZ. The remaining 2.5 mg of D were equally divided and added to each dart (0.415 mg of D per dart). The final solution contained 0.83 mg of D and 83.3 mg of TZ in each dart, with a D:TZ ratio of 1:100. For two-year-old bears, we prepared both drug combinations as described for yearlings, but divided the initial solution of M or D and TZ, and the remaining M or D into four 3 ml darts. The final solution contained 2.5 mg M or 1.25 mg D and 125 mg TZ in each dart, again with a M:TZ ratio of 1:50, and a D:TZ ratio of 1:100. The dose for each age class remained unchanged throughout the study.

We administered the anesthetic combination by remote delivery from a CO_2_-powered rifle (Dan-Inject^®^, Børkop, Denmark) at a distance of 3–7 meters from a helicopter. Darts used in the study consisted of 1.5 ml syringes with 1.5x25mm barbed needles with side ports (Dan-Inject^®^) in yearlings, and 3 ml syringes with 2.0x30mm needles in two-year-old bears. When needed, 1–2 mg/kg of ketamine (Narketan^®^ 100 mg/ml, Chassot, Dublin, Ireland) was administered intravenously or intramuscularly by syringe and needle to extend the duration of anesthesia.

The time intervals in minutes (min) from when a bear was first observed to when a bear was hit by a drug-filled dart (observed-darted time), from when active pursuit with the helicopter began to when the bear was darted (chased-darted time), and from when a bear was darted to recumbency (induction time) were recorded. We recorded capillary refill time (seconds), respiratory rate (breaths per min), heart rate (beats per min) and rectal temperature (°C) of anesthetized bears immediately after induction and every 15 min throughout the duration of anesthesia. Respiratory rate was monitored by observation of thoracic movements and heart rate by auscultation of the heart. Rectal temperature was measured with a digital thermometer (Accutemp^®^, Jahpron Medical Int., Jensvoll, Norway). When hyperthermia (≥ 40°C) occurred, we applied snow to the paws, groin and axillae, and administered intravenous fluids to reduce body temperature.

We collected one venous blood sample (8 ml) from the jugular vein of each bear as early as possible following induction using a vacutainer system (BD Vacutainer^®^, BD Diagnostics, Preanalytical Systems, Franklin Lakes, NJ, USA). We measured serum cortisol concentration (nmol/L) with this sample [30]. We also collected two anaerobic arterial blood samples (3 ml each) from the femoral artery of each bear in pre-heparinized syringes (PICOTM70, Radiometer Copenhagen, DK-2700 Brønshøj, Denmark), the first at 30 min, and the second at 60 min, after the bear was darted. We measured blood gases, acid-base status and selected hematologic and biochemical variables on site using a portable analyzer (iSTAT 1^®^Portable Clinical Analyzer and i-STAT^®^ cartridges CG4+ and 6+, Abbott Laboratories, Abbott Park IL, 60064–6048, USA). The parameters included pH, partial pressure of arterial carbon dioxide (PaCO_2_; mmHg), partial pressure of arterial oxygen (PaO_2_; mmHg), base excess (BE; mmol/L), bicarbonate (HCO_3_; mmol/L), total carbon dioxide (TCO_2_; mmol/L), arterial oxygen saturation (SaO_2_; %), lactate (mmol/L), sodium (mmol/L), chloride (mmol/L), potassium (mmol/L), blood urea nitrogen (BUN; mg/dL), glucose (mmol/L), hematocrit (% packed cell volume), and hemoglobin (g/dL). Blood gas values and pH were corrected to the rectal temperature.

Bears are routinely supplemented with intranasal oxygen throughout anesthesia, as part of the standard field procedure of the SBBRP [31]. However, for this study, we only administered oxygen to bears with low levels of blood oxygen (hypoxemia) based on PaO_2_ measurements. Below 80 mmHg, we considered bears to be hypoxemic and administered oxygen at a flow rate of 0.5 L/min in yearlings and 1L/min in two-year-old bears [29].

We performed different types of surgery (i.e., abdominal, muscle biopsy) on selected bears to meet the research objectives of other studies. In bears undergoing surgery, we preemptively administered 0.2 mg/kg of meloxicam (Metacam^®^ 5 mg/ml, Boehringer Ingelheim, Reihn, Germany) subcutaneously to reduce pain and inflammation caused by the surgery. We followed a standard protocol for other sampling and handling procedures [31]. Body weight was obtained by suspending bears from a spring-loaded scale to accurately determine drug dose levels (mg/kg of body weight).

After completion of all procedures, we administered 5 mg of atipamezole (Antisedan^®^ 5 mg/ml, Orion Pharma Animal Health) per mg of M or 10 mg of atipamezole per mg of D intramuscularly to reverse anesthesia. We recorded the time interval in min from recumbency to atipamezole administration (handling time), and left bears to recover undisturbed at the site of capture.

Brown bear captures occurred both on private and public lands. Captures were approved by the Swedish Ethical Committee on Animal Research (application numbers C 7/12 and C 18/15) and the Swedish Environmental Protection Agency (NV-0758-14).

### fRI Research Grizzly Bear Program (fRI)

We captured six free-ranging adult (6–15 years) male brown bears in western Alberta, Canada (52.865360–54.368277 N, 117.865738–119.017687 E) in May 2014–2015 by barrel (culvert) trap [32]. We applied a randomized, double-blinded study in which three bears were allocated to the MTZ group and three to the DTZ group. We prepared MZT by adding 12 mg of M (20 mg/ml; Chiron Compounding Pharmacy Inc., Guelph, Ontario, Canada) and 0.9 ml of sterile water for injection (Hospira 10 ml per vial, Montreal, Quebec, Canada) per vial of TZ (Telazol^®^, 286 mg tiletamine + 286 mg zolazepam; Fort Dodge Laboratories, Inc., Fort Dodge, Iowa, U.S.A.). DZT was prepared in 2014 by adding 5.7 mg of D (3 mg/ml; Chiron Compounding Pharmacy Inc.) and 0.2 ml of sterile water for injection per vial of Telazol^®^. In 2015, we used 6 mg of a higher concentration of D (5 mg/ml), plus 0.9 ml of sterile water for injection, per vial of Telazol^®^. All formulations resulted in 2.5 ml of drug solution per vial with concentrations of 234 mg/ml for MTZ and 231 mg/ml for DTZ, and ratios of 1:48 for M:TZ and 1:95 for D:TZ.

We used a remote drug delivery system (Dan-Inject^®^) to administer a combination of 50μg/kg estimated body weight of M, or 25μg/kg of D, and 2.45 mg/kg of TZ intramuscularly. Darts used in the study consisted of 3 ml syringes with 2.0x40mm barbed needles (Dan-Inject^®^). When necessary, we administered ketamine at 2 mg/kg (200 mg/ml; Chiron Compounding Pharmacy Inc.) intramuscularly by syringe and needle to extend the duration of anesthesia.

We recorded the induction time for each bear. Capillary refill time, respiratory rate, heart rate, and rectal temperature of anesthetized bears were obtained immediately after induction and every 15 min throughout anesthesia. Respiratory rate was monitored by observation of thoracic movements. We recorded pulse rate and hemoglobin oxygen saturation (SpO_2_; %) with a pulse oximeter (Nellcor NPB-40, Nellcor, Pleasanton, California, U.S.A). Rectal temperature was measured with a digital thermometer (Adtemp V Fast Read Pen Type Digital Thermometer, American Diagnostic Corporation, New York, U.S.A).

We collected one venous blood sample (4 ml) from the femoral vein of each bear to measure cortisol concentrations (nmol/L; Immulite 1000; Siemens Medical Solutions Diagnostics, California, U.S.A). We also collected two anaerobic arterial blood samples (3ml each) from the femoral artery of each bear in pre-heparinized syringes 30 and 60 min after the bear was darted. We used the same equipment and measured the same parameters as previously described. Blood gas values and pH were corrected to the rectal temperature. Although oxygen was available, we did not administer it to any of the bears captured in Alberta, Canada.

We extracted a premolar tooth for age estimation by counting cementum annuli [33]. We administered 0.1 mg/kg of meloxicam (Metacam^®^, 5mg/ml solution for injection; Boehringer Ingelheim Vetmedica Inc., Missouri, U.S.A) subcutaneously to provide analgesia. We weighed all bears with an electronic load-cell scale.

After completion of measurements and sampling, we administered 5 mg of atipamezole (20 mg/ml; Chiron Compounding Pharmacy Inc.) per mg of M or 10 mg of atipamezole per mg of D intramuscularly for anesthetic reversal. Bears were left to recover from anesthesia undisturbed at the site of capture. We recorded the handling time, and the time interval from atipamezole administration until the bear showed the first signs of recovery (recovery time, in min).

Brown bear captures were authorized under the permitting authority of the Alberta Department of Environment and Sustainable Resource Development (provincial jurisdiction lands), Alberta Tourism and Parks (provincial parks and protected areas jurisdiction lands), and Parks Canada (federal jurisdiction lands). Captures were approved by the University of Saskatchewan’s Committee on Animal Care and Supply (Animal Use Protocol # 20010016) and were in accordance with guidelines provided by the American Society of Mammalogists’ Animal Care and Use Committee [3] and the Canadian Council on Animal Care for the safe handling of wildlife [34].

### Statistical analysis

We approached the statistical analyses in three sequential phases, data exploration, model development, and model validation, using the software R 3.1.0 [35]. For data exploration, we evaluated the raw data for (i) missing values, (ii) presence of outliers, (iii) collinearity among potential predictor (independent) variables, and (iv) relationships or associations between response (dependent) and predictor variables [36]. We used mean values to substitute for missing values (i.e., we substituted two missing induction times when used as predictors with the mean value). Collinearity among predictor variables was evaluated by variance inflation factors (VIF ≥ 3.0) and pairwise correlations (r ≥ 0.7). Collinear variables were not used together in the same model. We standardized continuous predictor variables (covariates) prior to model development to facilitate comparisons among different models [37].

For model development, we worked with two different data sets. The first, containing data collected in Sweden only, and the second, combined datasets containing data collected both in Sweden and Alberta. We carried out different analyses for each of the hypotheses to be tested (Table 1). For the induction time, the need for supplemental drugs and stress hypotheses (i.e. Hypotheses 1–3), we used the ‘dredge’ function in package *MuMin* [38] to build all possible models containing a maximum of 3 (Swedish dataset) or 4 (combined datasets) predictor variables to avoid model overfitting. With the same goal, we also did not evaluate possible interactions. Model selection was based on the Akaike’s Information Criterion (AIC) [39]. For evaluation of the arterial blood gases and acid-base status, and physiological responses hypotheses (i.e. Hypothesis 4 and 5), we build multiple global models for each response variable to avoid collinearity (i.e., predictor collinear variables were not used together in the same model). We selected the most parsimonious (based on AIC) of these models for further analysis. Then we applied the ‘drop 1’ function [40] to obtain the final model. However, before dropping a predictor variable, we also evaluated it for any two-way interactions of potential physiological significance, e.g., drug combination x respiratory rate.

**Table 1:** Response and predictor variables (interactions not shown), model types, and sample sizes (N) used to test hypotheses in brown bears anesthetized with either medetomidine-tiletamine-zolazepam (MTZ) or dexmedetomidine-tiletamine-zolazepam (DTZ) in Sweden (S, N = 34) and Alberta, Canada (A, N = 6) in 2014–2015.

Hypotheses	Response variable^a^	Predictor variable^b^ combinations	Random effects^c^	Model type^d^	N
1	Induction time	Age + Sex + Drug + TZ + CD time + ODC^e^	NA	GLM Gamma link inverse	S = 34, S+A = 38
2	Supplemental drugs	Age + Sex + Drug + Weight + CD time + ODC + Induction time + Surgery + Handling time^e^	NA	GLM binomial	S = 34, S+A = 40
3	Cortisol	Age + Sex + Drug + Weight + CD time + ODC + Induction time^e^^,^^f^	NA	GLM Gaussian	S = 34, S+A = 39
4	pH	Time + Age + Drug + PaCO_2_ + BE + Lactate	Bear ID	LMM	S = 64, S+A = 76
4	PaO_2_	Age + Drug + Length + RT + RR + Oxygen	Bear ID	LMM	S = 64, S+A = 76
4	PaCO_2_	Age + Drug + Weight + RT + RR + PaO_2_	Bear ID	LMM	S = 64, S+A = 76
5	Heart rate	Time + Age + Sex + Drug + Length + CD time + ODC + Induction time + Surgery + Ket + RT + RR^e^	Bear ID	LMM	S = 223, S+A = 165
5	Respiratory rate	Time + Age + Sex + Drug + Length + CD time + ODC + Induction time + Surgery + Ket + RT + HR^e^^,^^f^	Bear ID	LMM	S = 224, S+A = 167
5	Rectal temperature	Time + Age + Sex + Drug + Weight + CD time + ODC + Induction time + Surgery + Ket + HR + RR^e^	Bear ID	LMM	S = 223, S+A = 165

^a^ Response variables—(i) Induction time: time interval in minutes from when a bear was darted to recumbency; (ii) Supplemental drugs: yes, no; (iii) Cortisol: serum concentration in nmol/L; (iv) pH: arterial blood acid-base status; (v) PaO_2_: partial pressure of arterial oxygen in mmHg; (vi) PaCO_2_: partial pressure of arterial carbon dioxide in mmHg; (vii) Heart rate (HR): beats per minute; (viii) Respiratory rate (RR): breaths per minute (log-transformed); and (ix) Rectal temperature (RT): °C.

^b^ Predictor variables—(i) Age: yearlings, two year olds, adults (≥5 yr); (ii) Sex: male, female; (iii) Drug: MTZ or DTZ in mg/kg body weight; (iv) TZ: tiletamine-zolazepam in mg/kg body weight; (v) CD time: time interval in minutes from when active pursuit began to when the bear was darted; (vi) ODC: ordinal day of capture; (vii) Weight: body weight in kg; (viii) Surgery: yes or no; (ix) Handling time: time interval in minutes from recumbency to atipamezole administration; (x) Area: Sweden, Alberta; (xi) PaCO_2_; (xii) Time: sampling and/or measurements recorded at 15; 30; 45; 60; 75; 90; 105; 120; 135 minutes after darting in Sweden, and at 15; 30; 45; 60; 75 minutes after darting in Sweden+Alberta; (xiii) BE: base excess in mmol/L; (xiv) Lactate: blood concentration in mmol/L; (xv) Length: contour body length in cm; (xvi) RR: respiratory rate; (xvii) RT: rectal temperature; (xviii) Oxygen: yes or no; (xiv) PaO_2;_ (xx); Ket: ketamine dose level in mg/kg body weight; (xxi) HR: heart rate; (xxii) RR: respiratory rate; (xxii) RT: rectal temperature

^c^ NA: not applicable.

^d^ GLM: generalized linear model; LMM: linear mixed model.

^e^ CD time was excluded as explanatory variable for the analysis of the Sweden+Alberta dataset.

^f^ Area (Sweden; Alberta) substituted age as explanatory variable for the analysis of the Sweden+Alberta dataset

For model validation, we plotted the standardized residuals of the best model against the fitted values to assess homogeneity. If a pattern was observed in the spread, we applied a transformation to the response variable.

We present the mean ± standard deviation for all variables, unless otherwise stated. Differences were considered significant when p ≤ 0.05.

## Results

### Hypothesis 1: The induction of anesthesia occurs faster with DTZ than MTZ

We used a single dart in the anesthesia of 30 bears (88%) in Sweden. Four bears (12%, two bears in each drug group) required an additional dart to achieve anesthesia. Bears allocated to the MTZ group (N = 16) received an average dose level of 93.62 ± 36.96 μg/kg M and 4.69 ± 1.85 mg/kg TZ. Bears in the DTZ group (N = 18) received an average dose level of 57.51 ± 38.37 μg/kg D and 4.87 ± 2.49 mg/kg TZ. Induction of anesthesia was quick (3.73 ± 2.81 min), predictable, and smooth in all bears irrespective of anesthetic protocol.

We used a single dart in the anesthesia of all bears captured by culvert trap in Alberta. Bears allocated to the MTZ group (N = 3) received an average dose level of 52.23 ± 18.55 μg/kg M and 2.5 ± 0.88 mg/kg TZ. Bears in the DTZ group (N = 3) received an average dose level of 21.97 ± 10.12 μg/kg D and 1.6 ± 0.78 mg/kg TZ. Induction of anesthesia was predictable and smooth in all bears irrespective of anesthetic protocol, but mean induction time was longer (6.25 ± 1.89 min) than recorded for bears in Sweden.

**Fig 1:**
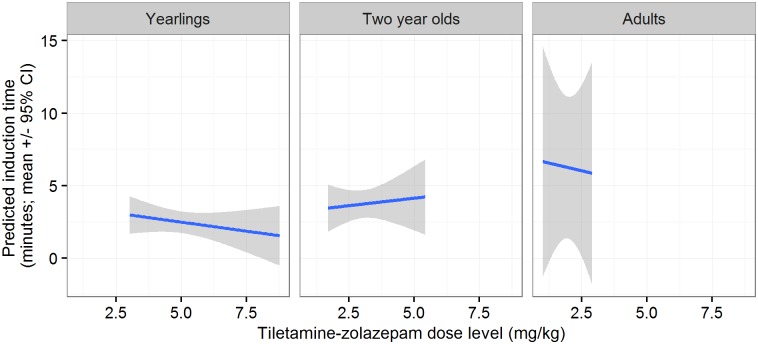
Induction time (time interval from when a bear was darted to recumbency, in minutes) by tiletamine-zolazepam dose level (in mg/kg body weight) and age class in 34 anesthetic events of free-ranging brown bears using a single dart of either medetomidine-tiletamine-zolazepam or dexmedetomidine-tiletamine-zolazepam in Sweden and Alberta, Canada in 2014–2015.

The induction time was significantly affected by TZ dose level, age, and sex (i.e., longer induction with increasing TZ dose level, in two-year-old bears, and in males) (Table 2). For the combined datasets, induction was faster in yearlings than in adult bears (Fig 1). Drug combination did not have a significant effect on induction time, and was not included in the best model. Thus, hypothesis 1 was not supported.

**Table 2:** Regression coefficients (β) and significance (p) of the predictor variables in the best model explaining variation in the response variables for hypotheses (H) 1, 2, 3 in brown bears anesthetized with either medetomidine-tiletamine-zolazepam (MTZ) or dexmedetomidine-tiletamine-zolazepam (DTZ) in Sweden (n = 34) and Alberta, Canada (n = 6) in 2014–2015.

	H1: Induction time				H2: Supplemental drugs				H3: Cortisol			
	Sweden		Sweden + Alberta		Sweden		Sweden + Alberta		Sweden		Sweden + Alberta	
Predictors^a^	β	p	β	p	β	p	β	p	β	p	β	p
Area (Sweden)											-369.59	0.034
Age (Yearlings)			0.286	<0.001			14.081	0.147				
Age (Two year olds)	-0.199	0.002	0.094	0.090			18.850	0.102				
Sex (Male)	-0.150	0.012	-0.145	0.012					134.03	0.007	104.99	0.037
TZ dose level	-0.051	<0.001	-0.049	<0.001								
Weight					4.947	0.054			-86.07	<0.001	-163.84	0.009
Ordinal day of capture					36.267	0.093	18.695	0.088			-43.17	0.071
Induction time									46.54	0.045		
Handling time					4.107	0.034	2.124	0.008				

^a^ Predictor variables–(i) Area: Sweden, Alberta; (ii) Age: yearlings, two year olds, adults (≥5 yr); (iii) Sex: male, female; (iv) TZ: tiletamine-zolazepam in mg/kg body weight; (v) Weight: body weight in kg; (vi) Induction time: time interval in minutes from when a bear was darted to recumbency; (vii) Handling time: time interval in minutes from recumbency to atipamezole administration. Regression coefficients for factors are relative coefficients such that: (i) β for Area (Sweden) was determined with β for Area (Alberta) set to 0; β for Age (Two year olds) was determined with β for Age (Yearlings) set to 0 for the Sweden dataset; (iii) β for Age (Yearlings) and for Age (Two year olds) were determined with β for Age (Adults) set to 0 for the Sweden + Alberta dataset; and (iv) β for Sex (Male) was determined with β for Sex (Female) set to 0.

### Hypothesis 2: The need for supplemental drugs to sustain anesthesia is lower with DTZ than MTZ

We administered supplemental drugs to extend anesthesia in 21 (62%) bears in Sweden. Of these, 11 bears belonged to the MTZ group, and 10 to the DTZ group. All bears but two received ketamine (1.81 ± 0.5 mg/kg) as the supplemental drug. Of these two bears, one showed signs of recovery 28 min after darting and received 2.55 mg/kg TZ. The other bear only received 2/3 of the DTZ dose when darted. So, the remaining 1/3 (15.22 μg/kg D and 1.49 mg/kg TZ) was administered when it showed signs of recovery 45 min after darting. We administered an average dose level of 2.22 mg/kg ketamine to extend anesthesia in two bears from the DTZ group in Alberta.

Handling time was the only variable that significantly influenced the need to administer additional drugs such that the longer the handling time, the greater the likelihood of using supplemental drugs to sustain anesthesia (Table 2). Because the need to administer supplemental drugs did not differ between DTZ and MTZ protocols, we did not find support for hypothesis 2.

### Hypothesis 3: Stress in response to capture and handling is lower with DTZ than MTZ

Among brown bears in Sweden, blood cortisol concentrations were significantly higher in bears that weighed less, in males, and in bears with longer inductions (Table 2). For the combined datasets, study area was also a determining factor. Cortisol concentrations were significantly higher in bears captured by culvert trap in Alberta than in bears captured by helicopter in Sweden (Table 2). Anesthetic protocol did not have a significant effect on cortisol levels. Therefore, hypothesis 3 was not supported.

### Hypothesis 4: Bears anesthetized with DTZ have higher pH and partial pressure of arterial oxygen (PaO_2_), and lower partial pressure of arterial carbon dioxide (PaCO_2_) than bears anesthetized with MTZ

We documented acidemia (pH < 7.35), hypoxemia (PaO_2_ < 80 mmHg), and hypercapnia (PaCO_2_ > 45 mmHg) as the main alterations in arterial blood gases and acid-base status using both anesthetic protocols and in both study areas (S1 Text).

Arterial blood pH decreased with PaCO_2_ values and increased with BE values in both datasets (Table 3). However, pH was not affected by drug protocol in either dataset. Thus, hypothesis 4 was not supported from the standpoint of our prediction that bears anesthetized with DTZ would have higher pH values than bears anesthetized with MTZ.

**Table 3:** Regression coefficients (β) and significance (p) of the predictor variables in the best model explaining variation in the response variables for hypothesis (H) 4 in brown bears anesthetized with either medetomidine-tiletamine-zolazepam (MTZ) or dexmedetomidine-tiletamine-zolazepam (DTZ) in Sweden (n = 34) and Alberta, Canada (n = 6) in 2014–2015.

	H4: pH				H4: PaO_2_				H4: PaCO_2_			
	Sweden		Sweden + Alberta		Sweden		Sweden + Alberta		Sweden		Sweden + Alberta	
Predictors^a^	β	p	β	p	β	p	β	p	β	p	β	p
Age (Yearlings)							-34.177	0.106				
Age (Two year olds)					18.560	0.029	-19.3013	0.242	6.597	0.004		
Sex (Male)												
Drug (MTZ)					1.628	0.704	2.903	0.449	0.926	0.363	0.398	0.730
Weight									-2.584	0.018		
Length					-8.181	0.044	-16.892	0.026				
Rectal temperature					-7.957	0.005	-6.478	0.004	-1.423	0.015	-0.715	0.231
Rectal temperature*MTZ					3.265	0.460			1.359	0.108	1.691	0.058
Respiratory rate					0.945	0.645	0.892	0.764	-1.867	0.001	-1.756	0.002
Respiratory rate*MTZ							0.326	0.928	2.078	0.004	0.662	0.006
PaCO_2_	-0.029	<0.001	-0.031	<0.001								
BE	0.058	<0.001	0.058	<0.001								
PaO_2_									1.755	<0.001	1.964	<0.001
Oxygen (Yes)					62.134	<0.001	62.288	<0.001				

^a^ Predictor variables–(i) Age: yearlings, two year olds, adults (≥5 yr); (ii) Sex: male, female; (iii) Drug: MTZ or DTZ in mg/kg body weight; (iv) Weight: body weight in kg; (v) Length: contour body length in cm; (vi) PaCO_2_: partial pressure of arterial carbon dioxide in mmHg; (vii) BE: base excess in mmol/L; (viii) Oxygen: supplementation with oxygen, yes, no. Regression coefficients for factors are relative coefficients such that: (i) β for Age (Two year olds) was determined with β for Age (Yearlings) set to 0 for the Sweden dataset; (ii) β for Age (Yearlings) and for Age (Two year olds) were determined with β for Age (Adults) set to 0 for the Sweden + Alberta dataset; (iii) β for Sex (Male) was determined with β for Sex (Female) set to 0; (iv) β for Drug (MTZ) was determined with β for Drug (DZT) set to 0; and (v) β for Oxygen (Yes) was determined with β for Oxygen (No) set to 0.

Arterial oxygen partial pressures (PaO_2_) were significantly correlated to the time interval from darting to sampling time (r = 0.75 in Sweden, r = 0.68 in the combined datasets, p < 0.001). The PaO_2_ values were higher in two-year-old bears in the Swedish dataset, but age class was not significant in the combined datasets (Table 3). Oxygen supplementation increased PaO_2_ values in the Sweden bears (Table 3). Although oxygen supplementation was also significant in the model describing the combined datasets, oxygen was not administered to bears in Alberta. Arterial oxygen partial pressures decreased with increasing body length and increasing rectal temperature in both datasets. However, PaO_2_ values were not affected by anesthetic protocol in either dataset (Table 3). Thus, hypothesis 4 was not supported from the standpoint of our prediction that bears anesthetized with DTZ would have higher PaO_2_ values than bears anesthetized with MTZ.

**Fig 2:**
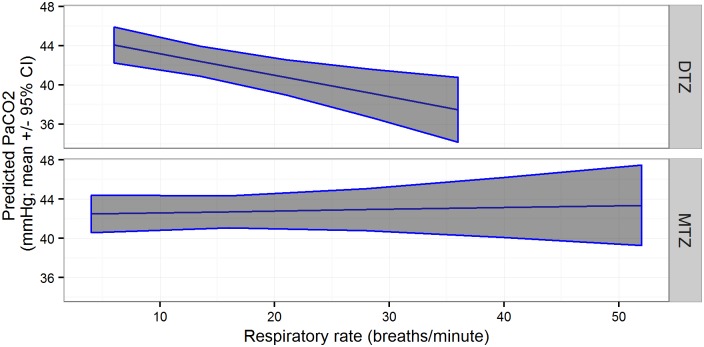
Partial pressure of arterial carbon dioxide (PaCO_2_, mmHg) by respiratory rate (breaths/minute) and drug combination (MTZ: medetomidine-tiletamine-zolazepam; DTZ: dexmedetomidine-tiletamine-zolazepam) in 40 anesthetic events of free-ranging brown bears captured in Sweden and Alberta, Canada in 2014–2015.

Arterial carbon dioxide partial pressures (PaCO_2_) were higher in two-year-old bears than yearlings, and decreased with increasing body weight and rectal temperature, in bears from Sweden, but these associations were not evident in the combined datasets (Table 3). There was a positive association between PaCO_2_ and PaO_2_ values, and a negative association between PaCO_2_ values and respiratory rates, in both datasets. The latter association was also significantly affected by anesthetic protocol in both datasets; PaCO_2_ values decreased as respiratory rate increased in the DTZ group, but remained relatively constant with changes in respiratory rate in the MTZ group (Table 3, Fig 2). Although not significant, there was a trend towards increasing PCO_2_ values with increasing rectal temperatures in the MTZ group in the combined datasets. These findings provide partial support for our prediction that bears anesthetized with DTZ would have lower PaCO_2_ values than bears anesthetized with MTZ, but this association was dependent on concurrent changes in respiratory rate. Overall, we found very little support for hypothesis 4.

### Hypothesis 5: DTZ produces less cardio-respiratory depression and quicker recovery of normal body temperature than MTZ

We detected bradycardia (< 50 beats per min), bradypnea (< 5 breaths per min), and hyperthermia (T ≥ 40°C) as the main physiological alterations during the anesthesia of bears with both anesthetic protocols. However, we observed differences between study areas (S2 Text).

Mean heart rate was lower in two-year-old bears than in yearlings among the Swedish bears, but this age class difference was not apparent in the model derived from the combined datasets (Table 4). Heart rate was positively associated with ordinal day of capture and with rectal temperature in both datasets. It was also positively associated with respiratory rate in both datasets, albeit non-significantly in the combined datasets (Table 4). Relative to heart rates recorded at 15 min following drug administration, heart rates in both datasets were generally lower at subsequent time points. Heart rate was not differentially affected by anesthetic protocol. Therefore, our prediction that DTZ would depress cardiovascular function (heart rate) less than MTZ was not supported.

**Table 4:** Regression coefficients (β) and significance (p) of the predictor variables in the best model explaining variation in the response variables for hypothesis (H) 5 in brown bears anesthetized with either medetomidine-tiletamine-zolazepam (MTZ) or dexmedetomidine-tiletamine-zolazepam (DTZ) in Sweden (n = 34) and Alberta, Canada (n = 6) in 2014–2015.

	H5: Heart rate				H5: Respiratory rate				H5: Rectal temperature			
	Sweden		Sweden + Alberta		Sweden		Sweden + Alberta		Sweden		Sweden + Alberta	
Predictors^a^	β	p	β	p	β	p	β	p	β	p	β	p
Area (Sweden)							0.644	<0.001				
Age (Yearlings)			37.415	0.092							0.529	0.161
Age (Two year olds)	-23.334	0.013	8.200	0.696	0.004	0.976					1.161	0.002
Sex (Male)	6.232	0.215	4.837	0.247								
Drug (MTZ)	-0.694	0.869										
Length	-5.948	0.142	0.620	0.946								
Length*Age (Yearlings)			-9.142	0.508								
Length*Age (Two year olds)			4.452	0.812								
CD time	4.043	0.096										
Ordinal day of capture	9.313	0.002	7.909	0.001								
Induction time	-4.40	0.242										
Induction time*Sex (Male)	6.903	0.153										
Surgery (Yes)	-1.824	0.718										
Ketamine dose level	-3.324	0.175	-3.280	0.121								
RT	5.134	<0.001	5.637	<0.001	-0.003	0.946						
RT*Age (Two year olds)					0.381	<0.001						
HR									0.370	<0.001	0.479	<0.001
RR	1.496	0.018	1.378	0.090								
Time (30 minutes)	-5.689	0.009	-2.985	0.154	-0.154	0.093			0.112	0.390	0.074	0.553
Time (45 minutes)	-8.032	<0.001	-5.374	0.009	-0.182	0.044			-0.005	0.969	-0.003	0.982
Time (60 minutes)	-7.205	0.002	-4.858	0.034	-0.148	0.119			-0.251	0.065	-0.234	0.083
Time (75 minutes)	-6.866	0.003	-5.780	0.029	0.047	0.616			-0.523	<0.001	-0.428	<0.001
Time (90 minutes)	-6.969	0.005			0.230	0.025			-0.695	<0.001		
Time (105 minutes)	-5.252	0.05			0.299	0.006			-0.966	<0.001		
Time (120 minutes)	-7-726	0.009			0.391	0.001			-1.024	<0.001		
Time (135 min)	-8.603	0.008			0.438	<0.001			-1.216	<0.001		

^a^ Predictor variables–(i) Area: Sweden, Alberta; (ii) Age: yearlings, two year olds, adults (≥5 yr); (iii) Sex: male, female; (vi) Drug: MTZ or DTZ in mg/kg body weight; (v) Length: contour body length in cm; (vi) CD time: time interval in minutes from when active pursuit began to when the bear was darted; (vii) Induction time: time interval in minutes from when a bear was darted to recumbency (viii) Surgery: yes or no; (ix) Ketamine dose level: in mg/kg body weight; (x) RT: rectal temperature; (xi) HR: heart rate; (xii) RR: respiratory rate; (xii) Time: minutes after darting when measurements were recorded. Regression coefficients for factors are relative coefficients such that: (i) β for Area (Sweden) was determined with β for Area (Alberta) set to 0; β for Age (Two year olds) was determined with β for Age (Yearlings) set to 0 for the Sweden dataset; (iii) β for Age (Yearlings) and for Age (Two year olds) were determined with β for Age (Adults) set to 0 for the Sweden + Alberta dataset; (iv) β for Sex (Male) was determined with β for Sex (Female) set to 0; (v) β for Drug (MTZ) was determined with β for Drug (DZT) set to 0; (vi) β for Surgery (Yes) was determined with β for Surgery (No) set to 0; and (vii) β for Times (30–135 minutes) were determined with β for Time (15 minutes) set to 0.

**Fig 3:**
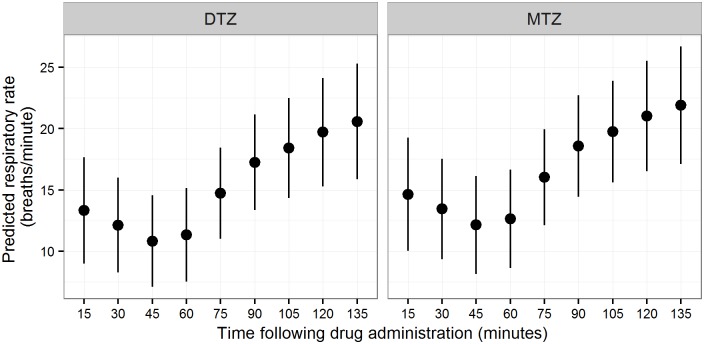
Respiratory rate (breaths/minute) over time following administration by drug combination (MTZ: medetomidine-tiletamine-zolazepam; DTZ: dexmedetomidine-tiletamine-zolazepam) in 34 anesthetic events of free-ranging brown bears captured in Sweden in 2014–2015.

Mean respiratory rate was significantly higher in bears captured by helicopter in Sweden than in bears captured by culvert trap in Alberta (Table 4). Respiratory rates were also affected by an interaction between rectal temperature and age in bears from Sweden (i.e., higher respiratory rates with increasing rectal temperatures in two-year-old bears), but this effect was not evident in the model derived from the combined datasets. Respiratory rates in bears from Sweden were significantly lower at 45 min than the first recording at 15 min following drug administration, and significantly higher at all time points from 90 to 135 min after drug administration. Respiratory rate was not differentially affected by anesthetic protocol (Fig 3). Therefore, our prediction that DTZ would produce less depression of the respiratory function (respiratory rate) than MTZ was not supported.

Rectal temperature was influenced positively by heart rate and negatively by time following drug administration. For the combined datasets, two-year-old bears had significantly higher rectal temperatures than adult bears (Table 4). Rectal temperature was not differentially affected by anesthetic protocol. Therefore, our prediction that MTZ would increase rectal temperature more than DTZ was not supported and, more generally, all three predictions under hypothesis 5 were not supported.

Atipamezole was used to end anesthesia in the two study areas (Sweden: 0.48 ± 0.21 mg/kg body weight, Alberta: 0.27 ± 0.1 mg/kg body weight). The duration of anesthesia (time interval from when a bear was darted to atipamezole administration) was longer in the bears captured in Sweden (132 ± 43 min) compared to Alberta (83 ± 25 min). The time interval from atipamezole administration until the bear showed the first signs of recovery was only documented in the bears captured with culvert trap in Alberta. Time of recovery was shorter in the DTZ group (median of 13 (8–26) min vs. 28 (26–54) min in the MTZ group) but, due to the small sample size, we did not perform a statistical analysis. No capture-related mortalities occurred in the study bears during or within one month following anesthesia as determined from movement data collected by GPS radio collars on study animals.

## Discussion

Both MTZ and DTZ proved to be safe and reliable drug combinations for anesthetizing free-ranging brown bears captured by helicopter and by culvert trap. However, we found no evidence to support use of DTZ as the better anesthetic combination. Both protocols produced a rapid onset of anesthesia, smooth induction, good analgesia and muscle relaxation, and smooth predictable recovery. Furthermore, the bears achieved an adequate plane of anesthesia for abdominal and subcutaneous surgeries, and muscle biopsies. We did not detect any bears’ reaction (i.e., increase in heart rate) to surgery.

Induction was smooth and adverse effects that could not be effectively treated were not encountered with either combination. The induction time in the study bears increased with an increasing dose level of TZ. This result could be explained since the bears receiving more than one dart (i.e., a higher dose level of the anesthetic combination) were the bears that took longer to achieve recumbency. When only bears anesthetized with a single dart were considered, the induction time decreased with an increasing dose level of TZ in yearlings and adults. This is in agreement with the results reported by Painer et al. (2012), where the length of the induction time in yearling brown bears anesthetized with one dart decreased with an increasing dose of M. In our study, we did not prove that induction occurs faster in bears receiving DTZ than MTZ. Therefore, we rejected our first hypothesis. Selmi et al. (2004) reported shorter times to initial sedative effects in golden-lion tamarins anesthetized with D-ketamine compared to M-ketamine. However, the same study found no difference in the time to lateral recumbency. In addition, the time from darting to first signs of sedation and recumbency were similar in Bennett’s wallabies (*Macropus rufogriseus*) and Chinese water deer (*Hydropotes inermis*) comparing two groups of animals receiving M-ketamine or D-ketamine [17, 18]. Although there are no previous comparisons of the effects of M and D in ursids, Teisberg et al. (2014) described induction times in bears captured with helicopter and anesthetized with DTZ similar to times found in studies using other drug combinations (xylazine-tiletamine-zolazepam, MTZ) [29, 41].

In accordance with previous studies in brown bears [42], the need for supplemental drugs to sustain anesthesia increased as the handling time increased. Using the same doses of MTZ for subadults and slightly lower doses for yearlings, Fahlman et al. (2011) reported that bears were sufficiently anesthetized to allow one hour of handling time. In our study in Sweden, the mean handling time was 128 ± 42 min, and supplemental drugs were necessary to sustain anesthesia in 62% of the bears. However, the need for supplemental drug administration was similar between anesthetic protocols. Thus, we rejected our second hypothesis. In wildlife species, a longer lasting anesthetic effect of D-ketamine over M-ketamine was discovered in golden-lion tamarins [16]. On the contrary, no difference in the duration of anesthesia was observed in wallabies and Chinese water deer at the time atipamezole was administered as reversal [17, 18]. Comparative studies between M and D have shown a longer lasting sedative effect of D in dogs and cats [13, 43]. Although, more recent studies have failed to prove any difference, and have concluded that M and D possess comparable sedative effects [44, 45].

Blood concentrations of cortisol, and glucose to a lesser extent, are widely-used parameters to assess the stress response to capture and handling in free-ranging wild animals [23, 24]. During the stress response to capture, glucocorticoid steroid hormones (including cortisol) are released into the blood circulation, and among their many effects is a sudden rise in blood glucose levels (i.e., hyperglycemia) [46]. Alpha-2 adrenoceptor agonists can reduce the stress of physical capture and handling due to their sedative effects (reduction of struggling and improvement of muscle relaxation) [47]. On the other hand, it is well documented that the use of alpha-2 adrenoceptor agonists increases plasma glucose concentrations through insulin release inhibition [26, 48]. The role of alpha-2 adrenoceptor agonists on cortisol concentrations is controversial, and varies among species [26, 27, 48–53]. Additionally, these studies suggest that the drug effect might be age and dose-dependent. Based on our results, we would suggest that bears with longer inductions, males, bears that weighed less, and bears captured by culvert trap vs. helicopter were more stressed by the capture event. However, blood cortisol concentrations did not support a lower stress response when using DTZ than when using MTZ, thus rejecting our third hypothesis. However, due to a paucity of information on the effect of alpha-2 adrenoceptor agonists, as well as TZ, in ursid species, caution should be taken. When drawing conclusions about capture-related stress by using cortisol concentrations in anesthetized animals, the potential for drug-induced effects should be considered.

We discovered acidemia (S3 Table) at similar levels to previous studies on brown bears captured by helicopter and anesthetized with MTZ in Scandinavia [29]. The reduction in pH values in our study can be attributed to a combination of respiratory and metabolic causes. The physical exertion during capture was probably responsible for acid lactic production and decrease of base excess values. This lead to a reduction in pH values due to metabolic acidosis in the early stages of the capture. A reduction in the respiratory rate due to the alpha-2 adrenoceptor agonists increased PaCO_2_ values causing respiratory acidosis. In our study, we rejected our fourth hypothesis as higher pH did not occur in bears anesthetized with DZT than MTZ.

We also documented hypoxemia (inadequate oxygen levels in the blood) which is a common physiological alteration found during the anesthesia of ursid species [7, 28, 29, 54]. The use of alpha-2 adrenoceptor agonists can cause respiratory depression and produce intrapulmonary changes that may result in hypoxemia [29, 55–57]. Hypoxemia can lead to hypoxia (inadequate oxygen levels in the body) that can have life-threatening consequences, such as myocardial ischemia, brain cell death and multi-organ damage [56, 58]. In the bears of the study, oxygen supplementation improved oxygenation and effectively treated hypoxemia as previously reported in brown bears [54, 59]. We found a decrease in PaO_2_ values with increasing rectal temperatures, as hyperthermia increases oxygen consumption [58]. Additionally, PaO_2_ values decreased with an increasing body length (significant correlated to dose level of alpha-2 adrenoceptor agonist). It is widely documented that effects of alpha-2 adrenoceptor agonists (i.e., sedation, analgesia, cardiovascular function) are dose-dependent [42, 55, 60, 61]. The alteration of the central and peripheral response to CO_2_ and oxygen is also dose-dependent [62]. A previous study in brown bears suggested that the hypoxemia caused by M could be dose-dependent [29]. Moreover, significantly lower PaO_2_ values were found when high doses of M and D were administered to dogs compared to lower doses [15]. Recently, studies using D in the anesthesia of bears found normal respiratory rates and high oxygen saturations [21, 22]. These authors suggested a potential benefit of D over M in bears due to less respiratory depression (i.e., hypoventilation, hypoxemia). However, these studies did not include a comparison of performance or efficacy with equivalent doses of M. In our study bears, contrary to Teisberg et al. (2014), both MTZ and DTZ caused hypoxemia (PaO_2_ < 80 mm Hg). We rejected our fourth hypothesis, as bears anesthetized with DTZ did not show higher PaO_2_ than bears anesthetized with MTZ. We argue that the different findings between Teisberg et al. (2014) and our study is due to the dose-dependent effect of alpha-2 adrenoceptor agonists on PaO_2_. The mean D dose level used in our study (21.97 ± 10.12 μg/kg in Alberta, 57.51 ± 38.37 μg/Kg in Sweden) was two to five times higher than in Teisberg et al. (2014) (10.11 ± 1.04 μg/Kg).

The hemoglobin oxygen saturation measured with pulse oximeter (SpO_2_) in the bears captured by culvert trap proved to be an unreliable indicator for hypoxemia in the study bears, as shown in other studies involving wildlife species [59, 63, 64]. For example, in one bear we measured 95% SpO_2_ that corresponded with PaO_2_ value of 59 mmHg recorded at the same point in time.

Values of PaCO_2_ represent the balance between cellular production of carbon dioxide (CO_2_) and ventilatory removal of CO_2_. CO_2_ elimination depends on the respiratory rate and the volume of inspired or expired air in one breath (tidal volume) [62]. Thus, we reported a reduction in PaCO_2_ caused by increasing respiratory rates. Nevertheless, hypercapnia was a more common physiological alteration documented in the study. PaCO_2_ values in our study were similar to previously reported values in brown bears anesthetized with MTZ in Scandinavia [29]. Mild to moderate hypercapnia may be beneficial in that it enhances the release of oxygen from hemoglobin into the tissues. However, severe hypercapnia, can lead to impaired myocardial contractility, narcosis, and coma [58]. PaCO_2_ values increased with increasing PaO_2_ values (correlated to time from darting to sampling time). Although provision of supplemental oxygen causes PaO_2_ values to increase, it has little effect on hypercapnia. The elevation of PaCO_2_ values usually indicates low respiratory rates (hypoventilation) that, in the study bears, was probably caused by the alpha-2 adrenoceptor agonists [29, 55]. In relation to PaCO_2_ values, we observed a differential effect of the anesthetic protocol. In the DTZ group, PaCO_2_ values decreased with increasing respiratory rates due to increased elimination of CO_2_. In contrast, PaCO_2_ values remained constant with increasing respiratory rates in the MZT group. Additionally, we found, although not significant, higher PaCO_2_ values with increasing rectal temperatures in the MTZ when data from Sweden and Alberta were combined. We believe that the greater variation in rectal temperature in the combined datasets was due to the different capture methods used, and therefore, made this interaction relevant. Furthermore, we believe that increasing rectal temperatures reflect increasing respiratory rates, as demonstrated in other studies with bears, where concurrent high respiratory rates and hyperthermia were documented [9, 29]. Surprisingly, these findings were not supported by significantly different respiratory rates between anesthetic protocols (i.e., higher respiratory rate in the DTZ group). Thus, we suggest that the results regarding PaCO_2_ values may be caused by a differential drug effect on the tidal volume (i.e., alveolar volume) and ventilation. The use of DTZ in the anesthesia of giant pandas (*Ailuropoda melanoleuca*) revealed changes in SpO_2_ with constant respiratory rates [19], supporting the fact that changes in ventilation might occur independently of respiratory rates. Anesthetic drugs can influence tidal volume by causing ventilation-perfusion problems [62]. Ventilation-perfusion problems lead to a decrease in PaO_2_ levels before any changes in PaCO_2_ levels. The administration of supplemental oxygen during anesthesia prevented us from detecting this effect. These results provide partial support to our fourth hypothesis that bears anesthetized with DTZ would have lower PaCO_2_ values than bears anesthetized with MTZ. We believe that D resulted in better ventilation than M, but only when respiratory rates increased. If this is true, D could prove more beneficial than M in situations when respiratory rates are anticipated to increase as in captures involving pursuit with a helicopter, captures with high ambient temperatures, or in later stages of anesthesia and during recovery. Nevertheless, we acknowledge that other comparative studies have not revealed differences between the use of M and the use of D on arterial blood gases and acid-base status [15, 17, 18].

In this study, mean heart rates remained within normal ranges (50–120 beats per min, S4 and S5 Tables) during the anesthetic period although we did observe bradycardia and tachycardia in some individual bears. Bradycardia secondary to vasoconstriction and hypertension is a common effect of the administration of alpha-2 adrenoceptor agonists [55, 65, 66]. Heart rates decreased over time as reported in previous studies [16, 20]. We also found lower heart rates in two-year-old bears than in yearlings in Sweden. Similarly, age differences have been previously reported in brown bears [29]. Brown bears in Scandinavia hibernate over a six-month period [67]. During this period, the bears do not eat, drink, defecate or urinate, and their metabolism is reduced. When bears emerge from the den after the hibernation period, their metabolic rate is approximately 50% of its normal rate which occurs sometime in the weeks following den emergence. For example, metabolic rate increased and stabilized 3 weeks following den emergence in black bears [68]. During this period of increased metabolism, heart rate, respiratory rate, body temperature, and movement rates increase [68, 69]. The bears of the study were captured from April, shortly after den emergence, to July. Thus, an increase in ordinal day of capture, accompanied by increasing rectal temperature and respiratory rate, would explain the increase in heart rate (used as an indicator of metabolic rate) [70]. We did not find fewer occurrences of bradycardia in bears receiving DTZ than in bears receiving MTZ. Therefore, we rejected our fifth hypothesis. Similarly, studies on other wildlife species have not found differences in the effect of M or D on heart rates [17, 18]. Selmi et al. (2004) showed that the heart rate in tamarins receiving D-ketamine was significantly lower than in the M-ketamine group. However, the authors attributed this result to different degrees of sedation and analgesia. In cats and dogs, numerous studies have reported contradictory results in comparing the effect of different doses of M and D on heart rate. For example, one study with domestic cats concluded that D and M have equivalent therapeutic effects [13], while another reported greater mean heart rates for M compared with D five min after drug administration, but mean heart rates were greater for D than for M at 180 min [44]. In dogs, Kuusela et al. (2001) reported a lower overall heart rate (area under the heart rate versus time) for D versus M in one of the dose levels (mg/kg) assessed but not in the others. These results suggest that the effects of alpha-2 adrenoceptor agonists on heart rates depend upon species, dose level and the time of measurement.

In this study, despite mean respiratory rates remaining within normal range (5–30 breaths/min) during anesthesia, hypoventilation likely occurred based on the magnitude of increases of PaCO_2_ values, and based on the respiratory rates reported in previous studies [29]. Similar to what has been reported in other studies, respiratory rate decreased over the first hour of anesthesia [16, 20, 29]. Respiratory rates increased after 90 min of anesthesia, probably due to a compensatory mechanism for hypercapnia and/or a light plane of anesthesia. We discovered higher respiratory rates in the Swedish bears than in the Alberta bears. This likely reflects the use of different captured methods, helicopter in Sweden vs. culvert trap in Alberta. Captures from helicopter often involve greater physical exertion with consequential increases in rectal temperature and respiratory rate prior to drug administration [71]. Bears receiving DTZ did not present lower respiratory rates than bears receiving MTZ. Hence, we rejected our fifth hypothesis. As previously mentioned, studies using D found normal respiratory rates during the anesthesia of bears [21, 22]. Bouts et al. (2011) also suggested that D would cause less respiratory depression compared to M. Nevertheless, studies in other wildlife species, as well as in domestic dogs and cats, have reported no differences in respiratory rates when comparing the two alpha-2 adrenoceptor agonists [13, 16, 17, 44, 45]. However, a study of laboratory mice reported higher respiratory rates in mice anesthetized with M-ketamine vs. D-ketamine [72].

We recorded body temperatures ≥ 40°C in the bears of the study. The highest body temperature recorded was 41.3°C in the MTZ group in Sweden. Hyperthermia has been previously reported in brown bears captured with helicopter [9, 29]. We found a significantly positive effect of age on rectal temperatures, two-year-old bears presented higher temperatures than yearlings and adult bears. This probably reflects the combined effect of a different capture method (helicopter in Sweden vs. culvert trap in Alberta) and the age difference among the bears of the two study areas (young bears in Sweden vs. adult bears in Alberta). Rectal temperatures in the Swedish bears were higher than in the Alberta bears due to physical exertion during helicopter pursuit [71]. Fahlman et al. (2011) reported lower rectal temperatures in yearling brown bears in comparison to subadults and adults. In our study, helicopter pursuit caused an increase in rectal temperature that masked the age effects on body temperature between yearlings in Sweden and adult bears captured in Alberta with culvert traps. Additionally, ambient temperature could also be an influencing factor as all yearlings were captured in April-May shortly after den emergence, while some two year olds were captured in July. Rectal temperatures significantly decreased over time in accordance with previous reports [18, 20, 29]. However, hypothermia was not observed at any time. The lowest body temperature recorded was 36.5°C in the DTZ group in Sweden. The alteration of thermoregulatory mechanisms by the alpha-2 adrenoceptor agonists [73], the cessation of physical activity, the onset of drug-induced muscle relaxation, and the application of corrective measures to reduce body temperature probably contributed to the decrease in body temperature [74]. Rectal temperature was not differentially affected by the drug combination used, hence, rejecting our fifth hypothesis that bears anesthetized with DTZ would show a quicker recovery of normal body temperature than MTZ. None of the studies comparing the effects of alpha-2 adrenoceptor agonists on thermoregulation in wildlife species have demonstrated any difference [16–18, 20]. However, these studies were performed in captive settings, where the animals were not subjected to high levels of physical exertion, and body temperatures were normal or close to normal at induction [17]. In free-ranging animals, especially those pursued during capture, we expect hyperthermia at early stages, irrespective of the anesthetic protocol used, due to stress and physical exertion. We also expect temperature to decrease and return to normal values over time. This decrease, however, might be affected by the anesthetic protocol used through the alteration of thermoregulatory mechanisms or changes in the respiratory rates [41, 75]. Drugs producing less depression of the respiratory function, would allow animals to better dissipate heat, and return to normal temperature values quicker [76]. In our study, we observed initial hyperthermia, and a decrease of rectal temperature over time as expected. Contrary to our hypothesis, both MZT and DZT produced the same level of respiratory depression on the bears, and therefore, no differences in rectal temperature between groups were detected at any time.

In Alberta, the time of recovery was shorter in the DTZ group. However, the dose level of atipamezole administered to the bears to reverse anesthesia was higher in the DZT (9.23 ± 1.08) than the MTZ group (7.85 ± 4.70). Furthermore, the sample size was small (n = 6). Thus, no definitive conclusions can be drawn. Results of previous studies in regards to recovery time are not in agreement. Some studies showed no difference in the recovery times [13, 72]. Other studies found a faster recovery with M than D when using a half-dose of atipamezole to reverse the effects of D [18]. Thus, the use of a full dose of atipamezole for D is recommended [18, 21]. When no reversal agent was used, Selmi et al. (2004) reported no differences in the time interval between the end of anesthesia and the animal standing, but longer times from standing until the animal could walk when using D in the anesthetic combination.

In summary, DZT and MZT produced reliable anesthesia without detectable differences in induction time, the need for supplemental drugs to sustain anesthesia, capture-related stress, acid-base status, PaO_2_, and physiological responses in free-ranging brown bears captured by helicopter or by culvert trap. DZT provided an apparent benefit by decreasing PaCO_2_ levels with increasing respiratory rates. However, this advantage was not supported by differential respiratory rates between anesthetic protocols. We recommend the use of supplemental oxygen to treat hypoxemia at the dose levels of alpha-2 adrenoceptor agonists used in the study. We conclude that dexmedetomidine offers no advantage over the use of medetomidine in the anesthesia of free-ranging brown bears when combined with tiletamine-zolazepam.

## Supporting Information

S1 Text Detailed results of pH, partial pressure of arterial oxygen (PaO_2_), and partial pressure of arterial carbon dioxide (PaCO_2_) in free-ranging brown bears (*Ursus arctos*) undergoing anesthesia with medeteomidine-tiletamine-zolazepam (MTZ) or dexmedeteomidine-tiletamine-zolazepam (DTZ) in Sweden (N = 34) and Alberta, Canada (N = 6) in 2014–2015. (DOCX)

S2 Text Detailed results of physiological responses in free-ranging brown bears (*Ursus arctos*) undergoing anesthesia with medeteomidine-tiletamine-zolazepam (MTZ) or dexmedeteomidine-tiletamine-zolazepam (DTZ) in Sweden (N = 34) and Alberta, Canada (N = 6) in 2014–2015. (DOCX)

S1 Table Capture date, age (years), sex (M: male; F: female), body weight (kg), body length (cm), drug combination used for anesthesia (DTZ: dexmedeteomidine-tiletamine-zolazepam; MTZ: medeteomidine-tiletamine-zolazepam), alpha-2 adrenoceptor agonist dose level (μg/kg), tiletamine-zolazepam dose level (TZ dose level, mg/kg), induction time (minutes), use and dose level of supplemental drugs (Suppl.drugs, Y: yes; N: no; Suppl. dose level, mg/kg) in 34 anesthetic events of free-ranging brown bears (*Ursus arctos*) captured in Sweden in 2014–2015. (DOCX)

S2 Table Capture date, age (years), sex (M: male; F: female), body weight (kg), body length (cm), drug combination used for anesthesia (DTZ: dexmedeteomidine-tiletamine-zolazepam; MTZ: medeteomidine-tiletamine-zolazepam), alpha-2 adrenoceptor agonist dose level (μg/kg), tiletamine-zolazepam dose level (TZ dose level, mg/kg), induction time (minutes), use and dose level of supplemental drugs (Suppl.drugs, Y: yes; N: no; Suppl. dose level, mg/kg) in six free-ranging brown bears (*Ursus arctos*) captured in Alberta, Canada in 2014–2015. (DOCX)

S3 Table Arterial blood gases, acid-base status, and oxygen saturation (mean ± standard deviation) in free-ranging brown bears (*Ursus arctos*) undergoing anesthesia with medeteomidine-tiletamine-zolazepam or dexmedeteomidine-tiletamine-zolazepam in Sweden (N = 34) and Alberta, Canada (N = 6) in 2014–2015. For the bears captured in Alberta, the median value and range are shown in parenthesis. Arterial blood gases and acid-base status were not measured in all bears at both sampling times.
(DOCX)

S4 Table Physiological responses (mean ± standard deviation) in 34 anesthetic events of free-ranging brown bears (*Ursus arctos*) using medeteomidine-tiletamine-zolazepam or dexmedeteomidine-tiletamine-zolazepam in Sweden in 2014–2015. Measurements were not recorded from all bears at all time points.
(DOCX)

S5 Table Physiological responses (mean ± standard deviation) in six free-ranging brown bears (*Ursus arctos*) undergoing anesthesia with medeteomidine-tiletamine-zolazepam or dexmedeteomidine-tiletamine-zolazepam in Alberta, Canada in 2014–2015. The median value and range are shown in parenthesis. Measurements were not recorded from all bears at all time points.
(DOCX)

S6 Table Hematological and biochemical parameters (mean ± standard deviation) in arterial blood from free-ranging brown bears (*Ursus arctos*) undergoing anesthesia with medeteomidine-tiletamine-zolazepam or dexmedeteomidine-tiletamine-zolazepam in Sweden (N = 34) and Alberta, Canada (N = 6) in 2014–2015. For the bears captured in Alberta, the median value and range are shown in parentheses. Blood parameters were not measured in all bears.
(DOCX)

## References

[1] Osofsky SA, Hirsch KJ. Chemical restraint of endangered mammals for conservation purposes: a practical primer. Oryx. 2000;34(1):27–33.

[2] Powell RA, Proulx G. Trapping and marking terrestrial mammals for research: integrating ethics, performance criteria, techniques, and common sense. ILAR J. 2003;44(4):259–276. 13130157 10.1093/ilar.44.4.259

[3] Sikes RS, Gannon WL. Guidelines of the American Society of Mammalogists for the use of wild mammals in research. J Mammal. 2011;92(1):235–253 10.1093/jmammal/gyw078 PMC5909806 29692469

[4] Kreeger TJ, Arnemo JM. Handbook of wildlife and chemical immobilization. 4^th^ ed Published by authors; 2012.

[5] Ramsay E. Ursidae and Hyaenidae In: Fowler ME, Miller RE, editors. Zoo and Wild Animal Medicine. 5^th^ ed Saunders Elsevier; 2003 pp. 523–528.

[6] Caulkett N, Fahlman Å. Ursids (Bears) In: West G, Heard G, Caulkett N, editors. Zoo Animal and Wildlife Immobilization and Anesthesia. John Wiley & Sons; 2014 pp. 599–606.

[7] Caulkett NA, Cattet MR, Caulkett JM, Polischuk SC. Comparative physiologic effects of Telazol^®^, medetomidine-ketamine, and medetomidine-Telazol^®^ in captive polar bears (*Ursus maritimus*). J Zoo Wildlife Med. 1999;30(4):504–509. 10749435

[8] Cattet MR, Caulkett NA, Lunn NJ. Anesthesia of polar bears using xylazine-zolazepam-tiletamine or zolazepam-tiletamine. J Wildlife Dis. 2003;39(3):655–664. 10.7589/0090-3558-39.3.655 14567228

[9] Taylor WP Jr, Reynolds HV III, Ballard WB. Immobilization of grizzly bears with tiletamine hydrochloride and zolazepam hydrochloride. J Wildlife Manage. 1989;53(4):978–981.

[10] Cattet MR, Caulkett NA, Polischuk SC, Ramsay MA. Reversible immobilization of free-ranging polar bears with medetomidine-zolazepam-tiletamine and atipamezole. J Wildlife Dis. 1997;33(3):611–617. 10.7589/0090-3558-33.3.611 9249708

[11] Savola JM, Virtanen R. Central α_2_-adrenoceptors are highly stereoselective for dexmedetomidine, the dextro enantiomer of medetomidine. Eur J Pharmacol. 1991;195(2):193–199. 1678707 10.1016/0014-2999(91)90535-x

[12] MacDonald E, Scheinin M, Scheinin H, Virtanen R. Comparison of the behavioral and neurochemical effects of the two optical enantiomers of medetomidine, a selective alpha-2-adrenoceptor agonist. J Pharmacol Exp Ther. 1991;259(2): 848–854. 1682487

[13] Ansah O, Raekallio M, Vainio O. Comparison of three doses of dexmedetomidine with medetomidine in cats following intramuscular administration. J Vet Pharmacol Ther. 1998;21: 380–387. 9811439 10.1046/j.1365-2885.1998.00155.x

[14] Jansson CC, Marjamäki A, Luomala K, Savola JM, Scheinin M, Åkerman KE. Coupling of human α_2_-adrenoceptor subtypes to regulation of cAMP production in transfected S115 cells. Eur J Pharm-Molec Ph. 1994;266(2):165–74. 10.1016/0922-4106(94)90106-6 7908883

[15] Kuusela E, Raekallio M, Väisänen M, Mykkänen K, Ropponen H, Vainio O. Comparison of medetomidine and dexmedetomidine as premedicants in dogs undergoing propofol-isoflurane anesthesia. Am J Vet Res. 2001;62(7):1073–1080. 11453483 10.2460/ajvr.2001.62.1073

[16] Selmi AL, Mendes GM, Figueiredo JP, Barbudo-Selmi GR, Lins BT. Comparison of medetomidine-ketamine and dexmedetomidine-ketamine anesthesia in golden-headed lion tamarins. Canadian Vet J. 2004;45(6):481–485. PMC548631 15283517

[17] Bouts T, Harrison N, Berry K, Taylor P, Routh A, Gasthuys F. Comparison of three anaesthetic protocols in Bennett’s wallabies (*Macropus rufogriseus*). Vet Anaesth Analg. 2010;37(3): 207–214. 10.1111/j.1467-2995.2009.00523.x 20230552

[18] Bouts T, Taylor P, Berry K, Routh A, Gasthuys F. Evaluation of medetomidine-ketamine and dexmedetomidine-ketamine in Chinese water deer (*Hydropotes inermis*). Vet Anaesth Analg. 2011;38(2):106–112. 10.1111/j.1467-2995.2010.00591.x 21303441

[19] Jin Y, Qiao Y, Liu X, Pu T, Xu H, Lin D. Immobilization of wild giant panda (*Ailuropoda melanoleuca*) with dexmedetomidine–tiletamine–zolazepam. Vet Anaesth Analg. 2015;43(3):333–337. 10.1111/vaa.12301 26332691

[20] Da Mota Lima CF, Cortopassi SRG, de Moura CA, de Mattos E Jr, d Candeias IZ, Pedron BG, et al Comparison between dexmedetomidine-s-ketamine and midazolam-s-ketamine in immobilization of oncilla (*Leopardus tigrinus*). J Zoo Wildlife Med. 2016;47(1):17–24. 10.1638/2013-0304.1 27010260

[21] Teisberg JE, Farley SD, Nelson OL, Hilderbrand GV, Madel MJ, Owen PA, et al Immobilization of grizzly bears (*Ursus arctos*) with dexmedetomidine, tiletamine, and zolazepam. J Wildlife Dis. 2014;50(1):74–83. 10.7589/2012-11-273 24171564

[22] Coltrane JA, Farley S, Saalfeld D, Battle D, Carnahan T, Teisberg J. Evaluation of dexmedetomidine, tiletamine, and zolazepam for the immobilization of black bears. Wildlife Soc B. 2015;39(2):378–382.

[23] Arnemo JM, Caulkett N. Stress In: West G, Heard D, Caulkett N, editors. Zoo Animal and Wildlife Immobilization and Anesthesia. Blackwell Publishing; 2008 pp. 103–109.

[24] Delehanty B, Boonstra R. Impact of live trapping on stress profiles of Richardson’s ground squirrel (*Spermophilus richardsonii*). Gen Comp Endocr. 2009;160(2):176–182. 10.1016/j.ygcen.2008.11.011 19059261

[25] Grimsrud K, Ait-Oudhia S, Durbin-Johnson B, Rocke D, Mama K, Rezende M., et al Pharmacokinetic and pharmacodynamic analysis comparing diverse effects of detomidine, medetomidine, and dexmedetomidine in the horse: a population analysis. J Vet Pharmacol Ther. 2015;38(1):24–34. 10.1111/jvp.12139 25073816 PMC4286451

[26] Arnemo JM, Ranheim B. Effects of medetomidine and atipamezole on serum glucose and cortisol levels in captive reindeer (*Rangifer tarandus tarandus*). Rangifer. 1999;19(2):85–89.

[27] Ranheim B, Horsberg T, Søli N, Ryeng K, Arnemo JM. The effects of medetomidine and its reversal with atipamezole on plasma glucose, cortisol and noradrenaline in cattle and sheep. Vet Pharmacol Ther. 2000;23(6):379–387. 10.1046/j.1365-2885.2000.00291.x 11168916

[28] Caulkett NA, Cattet MR. Physiological effects of medetomidine-zolazepam-tiletamine immobilization in black bears. J Wildlife Dis. 1997;33(3):618–622. 10.7589/0090-3558-33.3.618 9249709

[29] Fahlman Å, Arnemo JM, Swenson JE, Pringle J, Brunberg S, Nyman G. Physiologic evaluation of capture and anesthesia with medetomidine-zolazepam-tiletamine in brown bears (*Ursus arctos*). J Zoo Wildlife Med. 2011;42(1):1–11. 10.1638/2008-0117.1 22946363

[30] Græsli AR, Fahlman Å, Evans AL, Bertelsen MF, Arnemo JM, Nielsen SS. Haematological and biochemical reference intervals for free-ranging brown bears (*Ursus arctos*) in Sweden. BMC Vet Res. 2014;10:183–191. 10.1186/s12917-014-0183-x 25139149 PMC4236794

[31] Arnemo JM, Evans AL, Fahlman Å. Biomedical protocols for free-ranging brown bears, gray wolves, wolverines and lynx. 2012. http://www1.nina.no/RovviltPub/pdf/Biomedical%20Protocols%20Carnivores%20March%202012.pdf

[32] Cattet MR, Boulanger J, Stenhouse GB, Powell RA, Reynolds-Hogland MJ. An evaluation of long-term capture effects in ursids: implications for wildlife welfare and research. J Mammal. 2008;89(4):973–990.

[33] Stoneberg RP, Jonkel CJ. Age determination of black bears by cementum layers. J Wildlife Manage. 1966;30(2):411–414.

[34] Canadian Council on Animal Care. Guidelines on the care and use of wildlife. 2003. http://www.ccac.ca/Documents/Standards/Guidelines/Wildlife.pdf

[35] R Development Core Team 2014 R: a language and environment for statistical computing. R Foundation for Statistical Computing, Vienna, Austria http://www.R-project.org

[36] Zuur AF, Ieno EN. Beginner’s Guide to Zero-Inflated Models with R. Highland Statistics Ltd; 2016.

[37] Zuur AF, Ieno EN, Walker NJ, Saveliev AA, Smith GM. Mixed Effects Models and Extensions in Ecology with R. Springer Science+Business Media LLC; 2009.

[38] Barton K. Multi-Model Inference. CRAN Version 1.14.0. 2015.

[39] Burnham KP, Anderson DR. Model selection and multimodel inference: a practical information-theoretic approach. 2^nd^ ed Springer; 2002.

[40] Bates D, Maechler M, Bolker B, Walker S. Linear mixed-effects model using Eigen and S4. R package version 1.1–8. 2015.

[41] Cattet MR, Caulkett NA, Stenhouse GB. Anesthesia of grizzly bears using xylazine-zolazepam-tiletamine or zolazepam-tiletamine. Ursus. 2003;14(1):88–93. 10.7589/0090-3558-39.3.655 14567228

[42] Painer J, Zedrosser A, Arnemo JM, Fahlman Å, Brunberg S, Segerström P, et al Effects of different doses of medetomidine and tiletamine–zolazepam on the duration of induction and immobilization in free-ranging yearling brown bears (*Ursus arctos*). Can J Zoolog. 2012;90(6):753–757.

[43] Kuusela E, Raekallio M, Anttila M, Falck I, Molsa S, Vainio O. Clinical effects and pharmacokinetics of medetomidine and its enantiomers in dogs. J Vet Pharmacol Ther. 2000;23(1):15–20. 10747239 10.1046/j.1365-2885.2000.00245.x

[44] Granholm M, McKusick BC, Westerholm FC, Aspegrén JC. Evaluation of the clinical efficacy and safety of dexmedetomidine or medetomidine in cats and their reversal with atipamezole. Vet Anaesth Analg. 2006;33(4):214–223. 10.1111/j.1467-2995.2005.00259.x 16764585

[45] Granholm M, McKusick B, Westerholm F, Aspegrén J. Evaluation of the clinical efficacy and safety of intramuscular and intravenous doses of dexmedetomidine and medetomidine in dogs and their reversal with atipamezole. Vet Rec: J Br Vet Assoc. 2007;160(26):891–897. 10.1136/vr.160.26.891 17602104

[46] Reeder DM, Kramer KM. Stress in free-ranging mammals: integrating physiology, ecology, and natural history. J Mammal. 2005;86(2):225–235.

[47] Cattet MR, Caulkett NA, Wilson C, Vandenbrink T, Brook RK. Intranasal administration of xylazine to reduce stress in elk captured by net gun. J Wildlife Dis. 2004;40(3):562–565. 10.7589/0090-3558-40.3.562 15465726

[48] Restitutti F, Raekallio M, Vainionpää M, Kuusela E, Vainio O. Plasma glucose, insulin, free fatty acids, lactate and cortisol concentrations in dexmedetomidine-sedated dogs with or without MK-467: a peripheral α-2 adrenoceptor antagonist. Vet J. 2012;193(2):481–485. 10.1016/j.tvjl.2011.12.010 22277719

[49] Benson GJ, Grubb TL, Neff-Davis C, Olson WA, Thurmon JC, Lindner DL, et al Perioperative stress response in the dog: effect of pre-emptive administration of medetomidine. Vet Surg. 2000; 29(1): 85–91. 10653498 10.1111/j.1532-950x.2000.00085.x

[50] Ko JC, Fox SM, Mandsager RE. Sedative and cardiorespiratory effects of medetomidine, medetomidine-butorphanol, and medetomidine-ketamine in dogs. J Am Vet Med Assoc. 2000;216(10):1578–1583. 10825944 10.2460/javma.2000.216.1578

[51] Kanda T, Hikasa Y. Neurohormonal and metabolic effects of medetomidine compared with xylazine in healthy cats. Can J Vet Res. 2008;72(3):278–286. 18505192 PMC2327246

[52] Carroll GL, Hartsfield SM, Champney TH, Geller SC, Martinez EA, Haley EL. Effect of medetomidine and its antagonism with atipamezole on stress-related hormones, metabolites, physiologic responses, sedation, and mechanical threshold in goats. Vet Anaesth Analg. 2005;32(3):147–157. 10.1111/j.1467-2995.2005.00187.x 15877661

[53] Ambrisko T, Hikasa Y. Neurohormonal and metabolic effects of medetomidine compared with xylazine in beagle dogs. Can J Vet Res. 2002;66(1):42–49. 11858648 PMC226981

[54] Fahlman Å, Pringle J, Arnemo JM, Swenson JE, Brunberg S, Nyman G. Treatment of hypoxemia during anesthesia of brown bears (*Ursus arctos*). J Zoo Wildlife Med. 2010;41(1):161–164. 10.1638/2009-0036.1 20722273

[55] Jalanka HH, Roeken BO. The use of medetomidine, medetomidine-ketamine combinations, and atipamezole in nondomestic mammals: a review. J Zoo Wildlife Med. 1990;21(3):259–282.

[56] Read MR. A review of alpha_2_ adrenoreceptor agonists and the development of hypoxemia in domestic and wild ruminants. J Zoo Wildlife Med. 2003;34(2):134–138. 10.1638/1042-7260(2003)034[0134:AROAAA]2.0.CO;2 12885129

[57] Fahlman Å, Arnemo JM, Persson J, Segerström P, Nyman G. Capture and medetomidine-ketamine anesthesia of free-ranging wolverines (*Gulo gulo*). J Wildlife Dis. 2008;44(1):133–142. 10.7589/0090-3558-44.1.133 18263828

[58] Fahlman Å. Oxygen therapy In: West G, Heard G, Caulkett N, editors. Zoo Animal and Wildlife Immobilization and Anesthesia. John Wiley & Sons; 2014 pp. 69–82.

[59] Fahlman Å, Arnemo JM, Pringle J, Nyman G. Oxygen supplementation in anesthetized brown bears (*Ursus arctos*)-how low can you go? J Wildlife Dis. 2014;50(3):574–581. 10.7589/2013-06-148 24807187

[60] Lemke K. Anticholinergics and sedatives In: Tranquilli WJ, Thurmon JC, Grimm KA, editors. Lumb and Jones' veterinary anesthesia and analgesia. 4^th^ ed Blackwell Publishing; 2007 pp. 203–240.

[61] West G, Heard D, Caulkett N. Zoo Animal and Wildlife Immobilization and Anesthesia. 1^st^ ed Blackwell Publishing; 2007.

[62] McDonell WN, Kerr CL. Respiratory system In: Tranquilli WJ, Thurmon JC, Grimm KA, editors. Lumb and Jones' veterinary anesthesia and analgesia. 4^th^ ed Blackwell Publishing; 2007 pp. 117–152.

[63] Cattet MR, Caulkett NA, Streib KA, Torske KE, Ramsay MA. Cardiopulmonary response of anesthetized polar bears to suspension by net and sling. J Wildlife Dis. 1999;35(3):548–556. 10.7589/0090-3558-35.3.548 10479090

[64] Mich PM, Wolfe LL, Sirochman TM, Sirochman MA, Davis TR, Lance WR, et al Evaluation of intramuscular butorphanol, azaperone, and medetomidine and nasal oxygen insufflation for the chemical immobilization of white-tailed deer, *Odocoileus virginianus*. J Zoo Wildlife Med. 2008;39(3):480–487. 10.1638/2007-0150.1 18817017

[65] Vainio O, Palmu L. Cardiovascular and respiratory effects of medetomidine in dogs and influence of anticholinergics. Acta Vet Scand. 1988;30(4):401–408. 10.1186/BF03548016 PMC8142219 2640776

[66] Vainio O. Introduction to the clinical pharmacology of medetomidine. Acta Vet Scand. 1988;85: 85–88. 2571283

[67] Friebe A, Swenson JE, Sandegren F. Denning chronology of female brown bears in central Sweden. Ursus. 2001;12:37–45.

[68] Tøien Ø, Blake J, Edgar DM, Grahn DA, Heller HC, Barnes BM. Hibernation in black bears: independence of metabolic suppression from body temperature. Science. 2011;331(6019):906–909. 10.1126/science.1199435 21330544

[69] Evans AL, Singh NJ, Friebe A, Arnemo JM, Laske T, Fröbert O. et al Drivers of hibernation in the brown bear. Front Zool. 2016;13(1):1–11. 26870151 10.1186/s12983-016-0140-6 PMC4750243

[70] Butler PJ, Green JA, Boyd I, Speakman J. Measuring metabolic rate in the field: the pros and cons of the doubly labelled water and heart rate methods. Funct Ecol. 2004;18(2):168–183.

[71] Cattet MR, Christison K, Caulkett NA, Stenhouse GB. Physiologic responses of grizzly bears to different methods of capture. J Wildlife Dis. 2003;39(3):649–654. 10.7589/0090-3558-39.3.649 14567227

[72] Burnside WM, Flecknell PA, Cameron AI, Thomas AA. A comparison of medetomidine and its active enantiomer dexmedetomidine when administered with ketamine in mice. BMC Vet Res. 2013;9(1):48–57. 23497612 10.1186/1746-6148-9-48 PMC3605306

[73] Virtanen R. Pharmacological profiles of medetomidine and its antagonist, atipamezole. Acta Vet Scand. 1988;85:29–37. 2571275

[74] Ozeki LM, Caulkett N, Stenhouse G, Arnemo JM, Fahlman Å. Effect of active cooling and α-2 adrenoceptor antagonism on core temperature in anesthetized brown bears (*Ursus arctos*). J Zoo Wildlife Med. 2015;46(2):279–285. 10.1638/2014-0052R.1 26056880

[75] White JTH, Oli MK, Leopold BD, Jacobson HA, Kasbohm JW. Field evaluation of Telazol^®^ and ketamine-xylazine for immobilizing black bears. Wildlife Soc B. 1996;24(3):521–527.

[76] Stirling I, Spencer C, Andriashek D. Immobilization of polar bears (*Ursus maritimus*) with Telazol^®^ in the Canadian Arctic. J Wildlife Dis. 1989;25(2):159–168. 10.7589/0090-3558-25.2.159 2716095

