# Supplementary material for: A Double-Blinded, Randomized Comparison of Medetomidine-Tiletamine-Zolazepam and Dexmedetomidine-Tiletamine-Zolazepam Anesthesia in Free-Ranging Brown Bears (Ursus Arctos)
Source: PLoS One. 2017 Jan 24;12(1):e0170764. doi: 10.1371/journal.pone.0170764 (PMC5261618; doi:10.1371/journal.pone.0170764)
Supplement: S1 Text — (DOCX) [file pone.0170764.s001.docx]

**pH**

Arterial blood gases and acid-base status were not measured in all bears at both sampling times. We obtained the first and second arterial blood samples at 32 ± 5 and 63 ± 5 min after drug administration from bears in Sweden. Acidemia (pH < 7.35) occurred in 28 bears at 30 min after darting (13 of 14 bears in the MTZ group, 15 of 16 bears in the DTZ group). These included two bears in the DTZ group with severe acidemia (pH < 7.25). After one hour of anesthesia, 27 bears had acidemia (13 of 16 bears in the MTZ group, 14 of 18 bears in the DTZ group).

We obtained the first and second arterial blood samples at 34 ± 6 and 60 ± 2 min after drug administration from six bears in Alberta. Acidemia occurred in two bears (one of three bears in each group) 30 min after darting, but was not detected in any of the bears at 60 min.

**PaO_2_**

We recorded hypoxemia (PaO_2_ < 80 mmHg) in 27 bears captured in Sweden (13 of 14 bears in the MTZ group, 14 of 16 bears in the DTZ) at 30 min following drug administration. Of these, 20 bears (11 of 14 bears in the MTZ group, nine of 16 bears in the DTZ) had mild hypoxemia (PaO_2_ from 60-80 mmHg), and seven bears (two bears in the MTZ group, five bears in the DTZ) had marked hypoxemia (PaO_2_ from 40-60 mmHg). All 27 bears were supplemented with oxygen. At 60 min, four of seven bears not receiving oxygen (two bears in each anesthetic protocol) were hypoxemic.

Hypoxemia occurred in all Alberta bears at both sampling times. We recorded mild hypoxemia in three bears (one bear in the MTZ group, two bears in the DTZ group) and marked hypoxemia in the other three bears (two bears in the MTZ group, one bear in the DTZ group) at 30 min following drug administration. The PaO_2_ values increased slightly over time in all bears except one without provision of oxygen. One hour following drug administration, hypoxemia was mild in four bears (two bears in each group), and marked in two bears (one bear in each group). Values of hemoglobin oxygen saturation readings recorded by pulse oximeter (SpO_2_) were below 90% with both drug combinations.

**PaCO_2_**

We documented hypocapnia (PaCO_2_ < 35 mmHg) in one of 14 bears that received MTZ at 30 min following drug administration in Sweden. We found mild hypercapnia (PaCO_2_ from 45-60 mmHg) in three bears at 30 min after darting (one of 14 bears in the MZT, two of 16 bears in the DTZ group), and in 10 bears (six of 16 bears in the MTZ group, four of 18 bears in the DTZ group) at one hour following drug administration. Hypercapnia was severe (PaCO_2_ > 60 mmHg) in one of the bears in the MTZ group.

With the Alberta bears, one of three bears in the MTZ group was hypocapnic at one hour following drug administration, while another bear in the MTZ group was mildly hypercapnic at both sampling times.
