# Supplementary material for: A Double-Blinded, Randomized Comparison of Medetomidine-Tiletamine-Zolazepam and Dexmedetomidine-Tiletamine-Zolazepam Anesthesia in Free-Ranging Brown Bears (Ursus Arctos)
Source: PLoS One. 2017 Jan 24;12(1):e0170764. doi: 10.1371/journal.pone.0170764 (PMC5261618; doi:10.1371/journal.pone.0170764)
Supplement: S2 Text — (DOCX) [file pone.0170764.s002.docx]

**Heart rate**

We detected bradycardia (< 50 beats per min) in three bears (one of 16 bears in the MTZ group, two of 18 bears in the DTZ group) at 75 min following drug administration in Sweden. Heart rates lower than 50 beats per min were sustained until the end of the anesthesia in the bear belonging to the MTZ group, but increased above this rate in the other two bears. We detected tachycardia (> 120 beats per min) in three bears (two in the MTZ, one in the DTZ group). The elevated heart rate persisted longest in the bear belonging to the DTZ group.

We did not detect tachycardia at any time in the bears captured using culvert traps in Alberta. However, bradycardia was detected in four bears (one of three bears in the MTZ group, all three bears in the DTZ group) as early as 15 min after drug administration, and sustained until the end of the anesthesia.

**Respiratory rate**

We detected bradypnea (< 5 breaths per min) in two of 16 bears in the MTZ group at various times following drug administration in Sweden. Tachypnea (> 30 breaths per min) occurred in eight bears (five of 16 in the MTZ group, three of 18 in DTZ group) during anesthesia.

Respiratory rates were within the normal range (5-30 breaths per min) throughout anesthesia in the bears captured by culvert trap in Alberta.

**Body temperature**

Hypothermia (T < 35°C) was not recorded at any time during anesthesia in the Swedish bears. However, hyperthermia (T ≥ 40°C) was recorded in bears receiving both drug combinations. Five bears within each drug group were hyperthermic at 30 min after darting, and two bears within each drug group were still hyperthermic at 60 min.

Rectal temperature was within the considered normal range (35-40°C) throughout anesthesia in the bears captured by culvert trap in Alberta.
