# Supplementary material for: A Double-Blinded, Randomized Comparison of Medetomidine-Tiletamine-Zolazepam and Dexmedetomidine-Tiletamine-Zolazepam Anesthesia in Free-Ranging Brown Bears (Ursus Arctos)
Source: PLoS One. 2017 Jan 24;12(1):e0170764. doi: 10.1371/journal.pone.0170764 (PMC5261618; doi:10.1371/journal.pone.0170764)
Supplement: S1 Table — (DOCX) [file pone.0170764.s003.docx]

| **Bear ID** | **Capture date** | **Age** | **Sex** | **Weight** | **Length** | **Drug combination** | **Alpha-2 dose level** | **TZ dose level** | **Induction** | **Suppl. drugs** | **Suppl. dose level** |
| --- | --- | --- | --- | --- | --- | --- | --- | --- | --- | --- | --- |
| 1 | 4/23/2014 | 2 | M | 31.5 | 116 | MTZ | 105.4 | 5.29 | 13 | N | 0 |
| 2 | 4/23/2014 | 2 | M | 39 | 117 | DTZ | 21.3 | 2.14 | 4 | N | 0 |
| 3 | 4/24/2014 | 2 | M | 56 | 131 | DTZ | 22.3 | 2.23 | 6 | Y | 1.61 |
| 4 | 4/24/2014 | 2 | M | 51.5 | 132 | MTZ | 48.5 | 2.43 | 1 | Y | 1.75 |
| 5 | 4/26/2014 | 1 | F | 14.5 | 90 | DTZ | 57.2 | 5.74 | 2 | Y | 2.07 |
| 6 | 4/26/2014 | 1 | M | 17 | 93 | MTZ | 97.6 | 4.9 | 2 | Y | 1.76 |
| 7 | 4/26/2014 | 1 | M | 18 | 92 | MTZ | 92.2 | 4.63 | 3 | N | 0 |
| 8 | 4/26/2014 | 1 | M | 16 | 82 | DTZ | 51.9 | 5.21 | 1 | N | 0 |
| 9 | 4/27/2014 | 1 | F | 15 | 90 | MTZ | 110.7 | 5.55 | 1 | N | 0 |
| 10 | 4/27/2014 | 1 | M | 15.5 | 91 | DTZ | 53.5 | 5.37 | 2 | N | 0 |
| 11 | 4/27/2014 | 1 | F | 22 | 100 | DTZ | 37.7 | 3.79 | 2 | Y | 2 |
| 12 | 4/27/2014 | 1 | F | 25 | 109 | DTZ | 33.2 | 3.33 | 2 | N | 0 |
| 13 | 4/27/2014 | 1 | M | 27 | 110 | MTZ | 61.5 | 3.09 | 3 | N | 0 |
| 14 | 4/28/2014 | 1 | M | 22 | 108 | DTZ | 37.7 | 3.79 | 2 | N | 0 |
| 15 | 4/28/2014 | 1 | M | 16 | 87 | MTZ | 103.8 | 5.21 | 7 | Y | 1 |
| 16 | 4/28/2014 | 1 | M | 19 | 92 | DTZ | 43.7 | 4.38 | 2 | N | 0 |
| 17 | 7/1/2014 | 2 | F | 49 | 121 | MTZ | 33.9 | 1.7 | 3 | Y | 2.04 |
| 18 | 7/1/2014 | 2 | M | 42 | 115 | MTZ | 59.5 | 2.98 | 4 | Y | 1.67 |
| 19 | 7/1/2014 | 2 | M | 40 | 115 | DTZ | 31.3 | 3.13 | 4 | Y | 1.25 |
| 20 | 7/2/2014 | 2 | F | 43 | 116 | MTZ | 58.1 | 2.91 | 2 | Y | 2.33 |
| 21 | 7/2/2014 | 2 | F | 33 | 115 | DTZ | 37.9 | 3.79 | 4 | Y | 3.03 |
| 5 | 4/22/2015 | 2 | F | 23 | 103 | DTZ | 54.3 | 5.43 | 4 | N | 0 |
| 8 | 4/23/2015 | 2 | M | 37 | 118 | MTZ | 67.6 | 3.38 | 5 | Y | 1.08 |
| 7 | 4/23/2015 | 2 | M | 41 | 119 | DTZ | 30.5 | 3.05 | 4 | Y | 0.85 |
| 22 | 4/23/2015 | 1 | F | 18 | 98 | MTZ | 92.2 | 4.63 | 2 | N | 0 |
| 23 | 4/23/2015 | 1 | F | 14 | 86 | DTZ | 118.6 | 11.9 | 10 | N | 0 |
| 24 | 4/24/2015 | 1 | M | 21 | 97 | DTZ | 79 | 7.93 | 6 | Y | 1.9 |
| 25 | 4/24/2015 | 1 | M | 20.5 | 102 | MTZ | 162 | 8.13 | 10 | Y | 1.95 |
| 26 | 5/15/2015 | 1 | F | 18.4 | 93.5 | DTZ | 60 | 3.02 | 3 | Y | 2.17 |
| 27 | 5/15/2015 | 1 | F | 13.2 | 87 | MTZ | 126 | 6.31 | 2 | Y | 1.89 |
| 28 | 5/16/2015 | 1 | F | 11 | 81 | MTZ | 151 | 7.57 | 2 | Y | 2.27 |
| 29 | 5/16/2015 | 1 | F | 9.5 | 83 | DTZ | 175 | 8.77 | 1 | Y | 2.63 |
| 30 | 5/16/2015 | 1 | F | 13 | 89 | MTZ | 128 | 6.41 | 2 | Y | 1.92 |
| 31 | 5/16/2015 | 1 | F | 18 | 95 | DTZ | 90 | 4.63 | 6 | Y | 1.38 |
