# Supplementary material for: A Double-Blinded, Randomized Comparison of Medetomidine-Tiletamine-Zolazepam and Dexmedetomidine-Tiletamine-Zolazepam Anesthesia in Free-Ranging Brown Bears (Ursus Arctos)
Source: PLoS One. 2017 Jan 24;12(1):e0170764. doi: 10.1371/journal.pone.0170764 (PMC5261618; doi:10.1371/journal.pone.0170764)
Supplement: S2 Table — (DOCX) [file pone.0170764.s004.docx]

| **Bear ID** | **Capture date** | **Age** | **Sex** | **Weight** | **Length** | **Drug combination** | **Alpha-2 dose level** | **TZ dose level** | **Induction** | **Suppl. drugs** | **Suppl. dose level** |
| --- | --- | --- | --- | --- | --- | --- | --- | --- | --- | --- | --- |
| 1 | 5/14/2014 | 6 | M | 169.6 | 198 | MTZ | 31 | 1.5 | 9 | N | 0 |
| 2 | 5/19/2014 | 9 | M | 222.2 | 209 | DTZ | 10 | 1 | 5 | Y | 2.7 |
| 3 | 5/14/2015 | 9 | M | 118.8 | 176 | DTZ | 26 | 2.5 | 5 | N | 0 |
| 4 | 5/14/2015 | 15 | M | 298.6 | 221 | MTZ | 60 | 2.9 | 6 | N | 0 |
| 5 | 5/18/2015 | 8 | M | 115.2 | 175 | DTZ | 29 | 1.4 | NR | Y | 1.74 |
| 6 | 5/18/2015 | 8 | M | 167.8 | 196 | MTZ | 66 | 3.1 | NR | N | 0 |

_NR: not recorded_
