# Supplementary material for: A Double-Blinded, Randomized Comparison of Medetomidine-Tiletamine-Zolazepam and Dexmedetomidine-Tiletamine-Zolazepam Anesthesia in Free-Ranging Brown Bears (Ursus Arctos)
Source: PLoS One. 2017 Jan 24;12(1):e0170764. doi: 10.1371/journal.pone.0170764 (PMC5261618; doi:10.1371/journal.pone.0170764)
Supplement: S3 Table — For the bears captured in Alberta, the median value and range are shown in parenthesis. Arterial blood gases and acid-base status were not measured in all bears at both sampling times. (DOCX) [file pone.0170764.s005.docx]

|  | **Minutes following drug administration** | | | | | | | |
| --- | --- | --- | --- | --- | --- | --- | --- | --- |
|  | **30 min** | | | | **60 min** | | | |
|  | **N** | **Sweden** | **N** | **Alberta** | **N** | **Sweden** | **N** | **Alberta** |
| pH | 30 | 7.30 ± 0.04 | 6 | 7.35 ± 0.03 (7.36 (7.30-7.38)) | 34 | 7.33 ± 0.042 | 6 | 7.36 ± 0.02 (7.36 (7.34-7.40)) |
| PaO_2_ | 30 | 70 ± 10 | 6 | 60 ± 7 (59 (52-70)) | 34 | 121 ± 33 | 6 | 66 ± 10 (67 (50-79)) |
| PaCO_2_ | 30 | 41 ± 4 | 6 | 42 ± 4 (41 (36-49)) | 34 | 44 ± 4 | 6 | 41 ± 5 (41 (33-49)) |
| HCO_3_ | 30 | 20 ± 3 | 6 | 23 ± 3 (22 (20-28)) | 34 | 23 ± 4 | 6 | 23 ± 3 (23 (20-28)) |
| BE | 30 | -5 ±4 | 6 | -2 ± 3 (-3 (-6-3)) | 34 | -2 ± 4 | 6 | -2 ± 3 (-2 (-5-3)) |
| TCO_2_ | 30 | 21 ± 3 | 6 | 24 ± 3 (23 (21-29)) | 34 | 24 ± 4 | 6 | 24 ± 3 (24 (21-29)) |
| SaO_2_ | 30 | 87 ± 6 | 6 | 87 ± 5 (88 (79-93)) | 34 | 95 ± 10 | 6 | 90 ± 6 (91 (77-95)) |
| Lac | 30 | 3.65 ± 2.13 | 6 | 1.2 ± 0.58 (1.1 (0.7-2.3)) | 33 | 1.72 ± 0.80 | 6 | 0.93 ± 0.49 (0.76 (0.6-1.9)) |
| SpO_2_ | NR | NR | 5 | 90 ± 4 (90 (85-97)) | NR | NR | 6 | 92 ± 7 (94 (78-96)) |

_N: Sample size; PaCO2: partial pressure of arterial carbon dioxide, in mm Hg; PaO2: partial pressure of arterial oxygen, in mm Hg; BE: base excess, in mmol/L; HCO3: bicarbonate, in mmol/L; TCO2: total carbon dioxide, in mmol/L; SaO2: arterial oxygen saturation, in %; Lac: lactate concentration, in mmol/L; SpO2: Oxygen saturation readings obtained with a pulse oximeter, in % (only in Alberta); NR: not recorded_
