# Supplementary material for: A Double-Blinded, Randomized Comparison of Medetomidine-Tiletamine-Zolazepam and Dexmedetomidine-Tiletamine-Zolazepam Anesthesia in Free-Ranging Brown Bears (Ursus Arctos)
Source: PLoS One. 2017 Jan 24;12(1):e0170764. doi: 10.1371/journal.pone.0170764 (PMC5261618; doi:10.1371/journal.pone.0170764)
Supplement: S4 Table — Measurements were not recorded from all bears at all time points. (DOCX) [file pone.0170764.s006.docx]

| **Time after darting** | **N** | **Heart rate (beats/minute)** | **N** | **Respiratory rate (breaths/minute)** | **N** | **Body temperature (°C)** |
| --- | --- | --- | --- | --- | --- | --- |
| 15 min | 16 | 94 ± 17 | 16 | 17 ± 5 | 16 | 39.4 ± 0.8 |
| 30 min | 31 | 88 ± 17 | 31 | 16 ± 13 | 31 | 39.5 ± 0.9 |
| 45 min | 34 | 84 ± 19 | 34 | 14 ± 8 | 34 | 39.4 ± 0.8 |
| 60 min | 30 | 84 ± 20 | 30 | 13 ± 6 | 29 | 39.1 ± 0.8 |
| 75 min | 34 | 82 ± 22 | 34 | 16 ± 7 | 34 | 38.8 ± 1.0 |
| 90 min | 25 | 83 ± 20 | 25 | 19 ± 7 | 25 | 38.8 ± 0.8 |
| 105 min | 25 | 86 ± 21 | 25 | 20 ± 7 | 24 | 38.5 ± 0.8 |
| 120 min | 17 | 82 ± 24 | 17 | 20 ± 6 | 17 | 38.5 ± 0.6 |
| 135 min | 14 | 77 ± 23 | 14 | 20 ± 6 | 14 | 38.2 ± 0.7 |

_N: Sample size_
