# Supplementary material for: A Double-Blinded, Randomized Comparison of Medetomidine-Tiletamine-Zolazepam and Dexmedetomidine-Tiletamine-Zolazepam Anesthesia in Free-Ranging Brown Bears (Ursus Arctos)
Source: PLoS One. 2017 Jan 24;12(1):e0170764. doi: 10.1371/journal.pone.0170764 (PMC5261618; doi:10.1371/journal.pone.0170764)
Supplement: S5 Table — The median value and range are shown in parenthesis. Measurements were not recorded from all bears at all time points. (DOCX) [file pone.0170764.s007.docx]

| **Time after darting** | **N** | **Heart rate (beats/min)** | **N** | **Respiratory rate (breaths/min)** | **N** | **Body temperature (°C)** |
| --- | --- | --- | --- | --- | --- | --- |
| 15 min | 4 | 52 ± 5 (51 (47-58)) | 4 | 7 ± 1 (6 (6-8)) | 4 | 38.2 ± 0.7 (38.1 (37.5-39.2)) |
| 30 min | 5 | 54 ± 8 (51 (45-64)) | 6 | 7 ± 1 (7 (5-9)) | 6 | 37.8 ± 0.9 (37.7 (36.7-39.4)) |
| 45 min | 6 | 54 ± 7 (53 (43-62)) | 6 | 8 ± 1 (8 (6-10)) | 6 | 37.8 ± 0.9 (37.7 (36.6-39.4)) |
| 60 min | 6 | 51 ± 9 (52 (40-65)) | 6 | 7 ± 2 (6 (5-10)) | 6 | 37.8 ± 1.3 (37.9 (35.7-39.7)) |
| 75 min | 6 | 51 ± 9 (45 (36-55)) | 6 | 7 ± 2 (8 (6-10)) | 6 | 37.8 ± 1.3 (36.8 (36.6-37.1)) |

_N: Sample size_
