# Supplementary material for: A Double-Blinded, Randomized Comparison of Medetomidine-Tiletamine-Zolazepam and Dexmedetomidine-Tiletamine-Zolazepam Anesthesia in Free-Ranging Brown Bears (Ursus Arctos)
Source: PLoS One. 2017 Jan 24;12(1):e0170764. doi: 10.1371/journal.pone.0170764 (PMC5261618; doi:10.1371/journal.pone.0170764)
Supplement: S6 Table — For the bears captured in Alberta, the median value and range are shown in parentheses. Blood parameters were not measured in all bears. (DOCX) [file pone.0170764.s008.docx]

|  | **N** | **Sweden** | **N** | **Alberta** |
| --- | --- | --- | --- | --- |
| Sodium (mmol/L) | 23 | 129 ± 3 | 6 | 138 ± 1 (138 (136-140)) |
| Potassium (mmol/L) | 22 | 3.9 ± 0.4 | 6 | 3.9 ± 0.8 (4.0 (2.6-4.7)) |
| Chloride (mmol/L) | 18 | 103 ± 3 | 4 | 109 ± 4 (110 (104-113) |
| Blood Urea Nitrogen (mg/dL) | 18 | 10 ± 10 | 4 | 30 ± 15 (27 (16-49)) |
| Glucose (mg/dL) | 20 | 133 ± 35 | 6 | 193 ± 26 (192 (154-224)) |
| Hematocrit (%PCV) | 23 | 39 ± 4 | 6 | 45 ± 1 (44 (43-47)) |
| Hemoglobin (g/dl) | 23 | 13.2 ± 1.3 | 6 | 15.2 ± 0.5 (15.1 (14.6-16.0)) |
| Cortisol (nmol/L) | 34 | 293 ± 158 | 5 | 249 ± 161 (337 (33-406)) |

_N: Sample size_
